# Impact of Lipid Composition and Receptor Conformation on the Spatio-temporal Organization of μ-Opioid Receptors in a Multi-component Plasma Membrane Model

**DOI:** 10.1371/journal.pcbi.1005240

**Published:** 2016-12-13

**Authors:** Kristen A. Marino, Diego Prada-Gracia, Davide Provasi, Marta Filizola

**Affiliations:** Department of Pharmacological Sciences, Icahn School of Medicine at Mount Sinai, New York, United States of America; Heidelberg Institute for Theoretical Studies (HITS gGmbH), GERMANY

## Abstract

The lipid composition of cell membranes has increasingly been recognized as playing an important role in the function of various membrane proteins, including G Protein-Coupled Receptors (GPCRs). For instance, experimental and computational evidence has pointed to lipids influencing receptor oligomerization directly, by physically interacting with the receptor, and/or indirectly, by altering the bulk properties of the membrane. While the exact role of oligomerization in the function of class A GPCRs such as the μ-opioid receptor (MOR) is still unclear, insight as to how these receptors oligomerize and the relevance of the lipid environment to this phenomenon is crucial to our understanding of receptor function. To examine the effect of lipids and different MOR conformations on receptor oligomerization we carried out extensive coarse-grained molecular dynamics simulations of crystal structures of inactive and/or activated MOR embedded in an idealized mammalian plasma membrane composed of 63 lipid types asymmetrically distributed across the two leaflets. The results of these simulations point, for the first time, to specific direct and indirect effects of the lipids, as well as the receptor conformation, on the spatio-temporal organization of MOR in the plasma membrane. While sphingomyelin-rich, high-order lipid regions near certain transmembrane (TM) helices of MOR induce an effective long-range attractive force on individual protomers, both long-range lipid order and interface formation are found to be conformation dependent, with a larger number of different interfaces formed by inactive MOR compared to active MOR.

## Author Summary

The μ-opioid receptor (MOR) is an important pharmaceutical target in the treatment of pain. In order to develop novel pain therapies, devoid of the serious side-effects of present opioid analgesics, we need to understand the fundamentals of how MOR works on the molecular level. While some studies suggest that oligomers of MOR could play a role in signaling, how MOR forms dimers, which interfaces form, and the exact role of oligomers in MOR function remain unclear. While research has shown that the membrane environment can affect membrane protein function, most previous computational work to study oligomerization has been performed in a very simple membrane. Here, we use molecular dynamics simulations of MOR in a heterogeneous plasma membrane model (comprising 63 lipid types) to investigate how the presence of the protein modulates its lipid environment, affecting species distribution and sculpting characteristic order and thickness profiles around the receptors. Such modulations, in turn, induce long-range interactions between the proteins and favor the formation of specific dimeric conformations.

## Introduction

Elucidating the impact of the lipid environment on membrane proteins, including G Protein-Coupled Receptors (GPCRs), is increasingly being recognized as a crucial part of understanding how these proteins function. Cholesterol (CHOL), the lipid for which the most is known about its effect on GPCRs, has been shown to affect receptor thermal stabilization [1,2], agonist affinity [3,4], and oligomerization [5–8]. The conformational equilibrium of the prototypic GPCR rhodopsin is known to be sensitive not only to CHOL levels, but also to phospholipid headgroup and chain saturation [9]. Lipid headgroup charges have also been shown to play a role in the function of the β_2_ adrenergic (β_2_AR) [10] and the neurotensin NTS1 receptors [11].

The role of the lipids has primarily been attributed to indirect effects such as changing the physical properties of the membrane (e.g., thickness, curvature, surface tension, and elastic properties). Transmembrane proteins frequently experience hydrophobic mismatch in which the lengths of the hydrophobic chains of the lipids and the hydrophobic part of the protein are different. To rectify this mismatch, the protein adopts several strategies, including conformational changes and remodeling of the membrane thickness by changing the order of the lipids [12]. On a larger scale, heterogeneous membranes are organized into domains which are either liquid-ordered (l_o_) or liquid-disordered (l_d_) regions. Notably, some of these domains (e.g., lipid rafts) are enriched in CHOL and sphingolipids, two lipids which have a high propensity of being ordered [13], i.e., parallel to the membrane normal. While the exact role of lipids rafts is debated, they appear to aid in lateral organization of the proteins in the membrane, increasing the propensity of the necessary components of a cell signaling complex to come together [13,14].

In addition to modulating the physical properties of the plasma membrane, lipids can interact directly with membrane proteins. Several crystal structures of GPCRs, including β_2_AR [2] and the adenosine A_2A_ receptor [15], have been crystallized with interacting CHOL, suggesting that these molecules are strongly bound to the protein. A conserved consensus CHOL binding motif (CCM) has been identified in a number of GPCRs [2], while a sphingolipid binding site has been proposed for the serotonin 5HT_1A_ receptor [16]. CHOL-receptor interactions have also been suggested to play a role in oligomerization based on inferences from crystal structures of GPCRs (e.g., the β_2_AR [17] and the metabotropic mGlu2 receptor [18]) showing CHOL molecules at putative dimeric interfaces.

Molecular dynamics (MD) simulations of membrane mimetic systems continue to complement experiments by offering a mechanistic understanding of the dynamics not readily available to most experimental techniques. Early coarse-grained (CG) simulations of rhodopsin embedded in bilayers of lipids with different chain lengths and saturation levels showed that the membrane deforms to adapt to the protein [19]. Activation of rhodopsin was also shown to be affected by the physical properties of the membrane [20]; while lipids with unsaturated chains promote activation, CHOL, which is a much more rigid molecule, inhibits activation. Furthermore, while unsaturated lipids were shown by MD to preferentially cluster at the receptor in a non-specific manner [21,22] putative binding sites were identified for CHOL, notwithstanding the typically dynamic interaction between CHOL and GPCRs [23–26]. Notably, MD simulations of β_2_AR performed with different concentrations of CHOL showed that CHOL binding to conserved sites could prevent some dimer interfaces from forming [6].

While most published MD simulations have been performed in a single or dual component membrane, an average idealized multi-component plasma membrane parameterized recently within the Martini CG force-field [27] offers an unprecedented opportunity to study the impact of lipid composition on the spatio-temporal organization of GPCRs in a more realistic environment. This 63-component membrane mimetic contains combinations of all major lipid headgroups with different fatty acid tails, asymmetrically distributed between two plasma leaflets (see Ref. [27] for more details). Specifically, the following lipid species were included: i) the charged species phosphatidylinositol (PI), phosphatidylserine (PS), and phosphatic acid (PA), phosphatidylinositol mono-, bis-, and tri-phosphate (PIP_1-3_), and gangliosides (GM); ii) the zwitterionic lipid species phosphatidylcholine (PC), phosphatidylethanolamine (PE), and sphingomyelin (SM), and iii) the minor species diacylglycerol (DAG), ceramide (CER), and lysophosphatidylcholine (LPC). Simulating receptor self-association in this membrane model provides further insight into how lipids can affect oligomerization, adding to what we had previously concluded from simulations of opioid receptors in an environment composed of 1-palmitoyl-2-oleoyl-sn-glycero-3-phosphocholine (POPC) and 10% CHOL [28].

The μ-opioid receptor (MOR) is an ideal test case to examine the role of lipids and receptor conformation on oligomerization. First and foremost, the crystal structures of both inactive [29] and activated [30] MOR have been solved with the latter showing the expected outward swing-out movement of TM6. While a crystallographic TM1,2,H8/TM1,2,H8 interface was observed in both active and inactive structures, a TM5,6/TM5,6 interface was identified in the crystal structure of the inactive receptor, but not in that of its activated form. Furthermore, although the exact role of MOR oligomerization in signaling is still under debate, the potential of developing new, more effective therapies in the treatment of pain [31] by selectively targeting MOR heterodimers makes these systems worthy of further investigation. Finally, while there is evidence both for [32] and against [33] the association of MOR with lipid rafts, CHOL has been shown to be important in the spatio-temporal signaling by MOR [34]. In fact, it has been shown to promote homodimerization of MOR [5], agonist binding [35], coupling with G-proteins [5,35], and translocation of β-arrestin [36].

Here, we present the results of CG MD simulations of arrays of 16 inactive and/or activated MORs in the aforementioned multi-component plasma membrane carried out to further evaluate the role of both lipid environment and protein conformation on MOR oligomerization.

## Results

### Lipid Distributions around Individual Receptors

**Fig 1:**
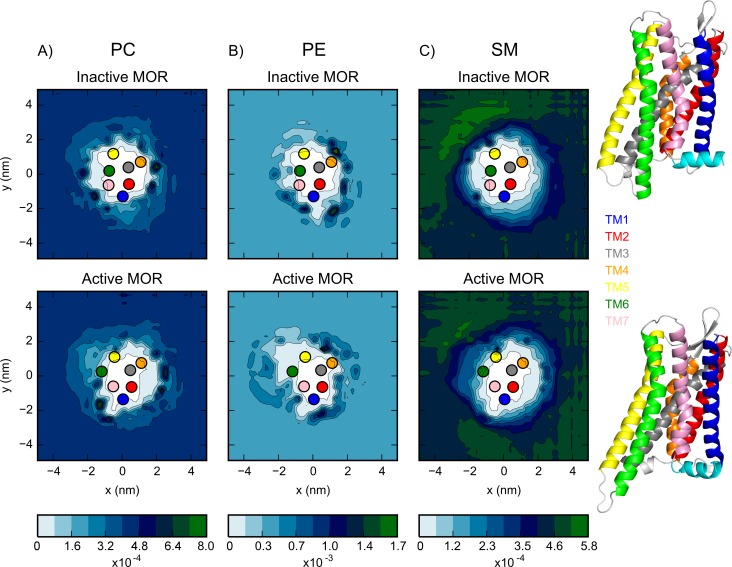
The normalized probability distribution of the A) PC, B) PE and C) SM lipids around the inactive and active receptor protomers (top and bottom panels, respectively) during the simulations with low receptor density and position restraints on the receptors. The center of mass of the seven TM helices are indicated by the colored dots as follows: TMs 1 through 7 are colored in blue, red, grey, orange, yellow, green, and pink, respectively. The helices of the crystal structures used as the starting structures are colored according to the same color scheme.

The 63 lipid types in the plasma membrane model were grouped by their headgroup to examine the preferred regions of association between the lipids and the receptors during simulation. Specifically, for this analysis, we used the final 2 μs of membrane equilibration of four sets of simulations: 16 inactive or active receptors in either a 50×50 nm^2^ or 25×25 nm^2^ membrane (referred to as low and high receptor density membranes, respectively). To analyze the behavior of the lipids, the GL1 or AM1 bead was used for all non-sterol lipids while the ROH bead was used for CHOL (See S1 Fig for a depiction of representative lipids). The normalized 2D probability distributions of lipids with zwitterionic headgroups (i.e., PC, PE, SM) in the simulations of the plasma membrane model with low density of inactive or active receptors are shown in Fig 1 whereas the distributions of all charged lipid types (i.e., GM, PS, PI, PIP_1-3_, and PA) are shown in S2 Fig. Unlike the distributions of PA, PI and PIP_1-3_, the distributions of PC, PE, SM, PS, or GM appear to be qualitatively similar around inactive and activated receptors. To confirm these observations, we calculated the standard deviation of the distribution of each lipid type at each grid point for five runs of inactive or active receptors, and compared it to the difference between the average lipid distributions around inactive and active receptors.

**Fig 2:**
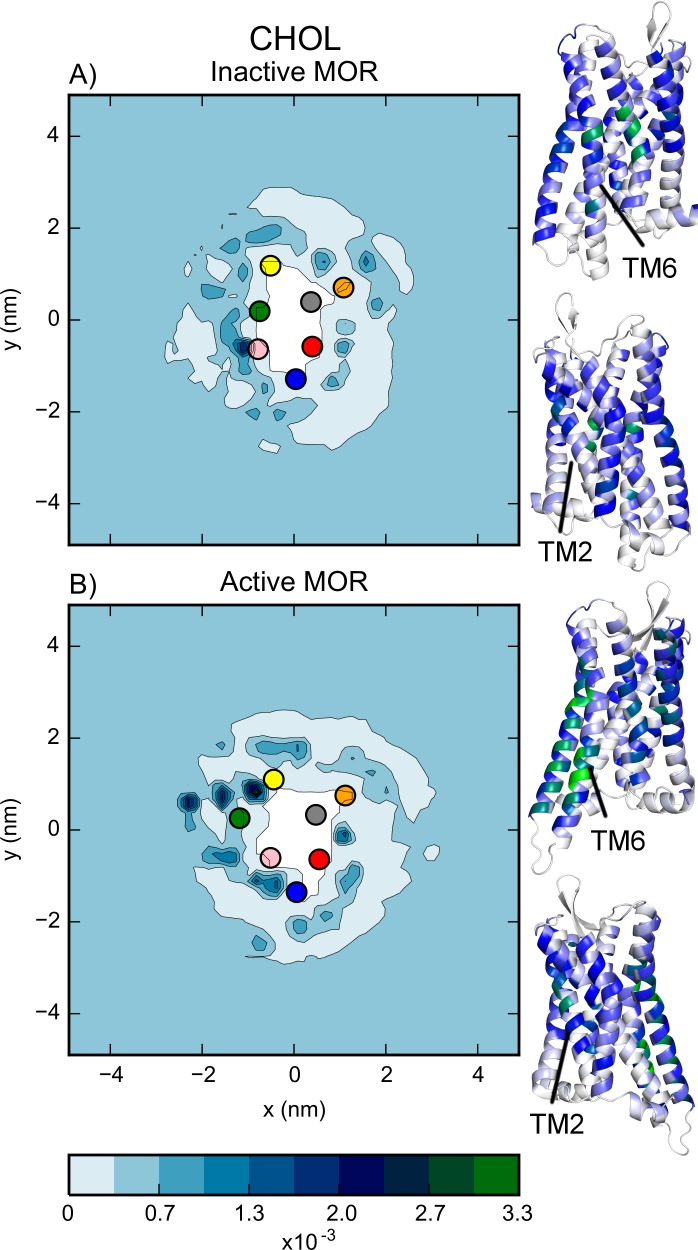
The normalized probability distribution of CHOL around A) inactive and B) active MOR protomers during the simulations with low receptor density and position restraints on the receptors. The center of mass of the seven TM helices are indicated by the colored dots as follows: TMs 1 through 7 are colored in blue, red, grey, orange, yellow, green, and pink, respectively. Also shown are structures of the inactive and active MOR with the residues colored by their probability of being in contact with the ROH bead of a CHOL (white to blue to green indicates low to high probability).

The distributions of PC and PE, which are the most populated glycerophospholipids in the plasma membrane, show a depletion of lipids immediately next to certain receptor TM helices. For instance, comparing Fig 1A and Fig 2, we observe that the lipid depletion at TM5 and TM6 (yellow and green dots in Fig 1A) is due to the presence of CHOL immediately next to the protein.

The remaining glycerophospholipids, PA, PI, PS, and PIP_1-3_, make up approximately 19% of the lower leaflet and are not present in the upper leaflet (See S3 Fig for the composition of the membrane). Despite their random initial position, these lipids diffuse towards the proteins during equilibration. Since there are a number of positively charged Lys (specifically, residue number 98, 100, 185^4.43^, 174, 260^5.66^, 269^6.24^, 271^6.26^, and 344) and Arg (i.e., 95^1.59^, 165^3.50^, 179, 182^4.40^, 258^5.64^, 263, 273^6.28^, 276^6.31^, 277^6.32^, 280^6.35^, 345, 348) residues on the intracellular side of MOR, it is not surprising to see PA, PI, PS, and PIP_1-3_, which are all negatively charged, localize in this region of the proteins (S2A–S2D Fig for PA, PI, PIP_1-3_ and PS, respectively). Specifically, PIP_1-3_ molecules are found to be predominantly in contact with the intracellular ends of TM4, TM6, and TM7 of inactive MOR. Upon activation, the outward swing of TM6 away from TM3 leaves the positively charged residues on TM5 and TM6 more exposed to negatively charged lipids, so that their concentration is reduced in the space between TM6 and TM7 and increased near TM5 and H8. See S1 Table for a list of the residues most frequently in contact with PIP_1-3_. Despite the small concentration of these PIP_1-3_ lipids in the membrane, the maximum difference between their distributions around inactive and active receptors (0.03) is much larger than the maximum standard deviation (0.005) of the PIP_1-3_ distribution at each grid point for the five runs of inactive or active receptors, suggesting that the observed conformation-dependent changes are larger than the sampling error. On the other hand, the PA and PI lipids (S2 Fig Panels A and B, respectively) do not show significant changes in distribution around the two receptor conformations, because the differences in lipid distribution around inactive and active receptors (PA: 0.003, PI: 0.006) are the same order of magnitude as the maximum standard deviation at each grid point (PA: 0.002, PI: 0.002).

In addition to the glycerophospholipids, the plasma membrane contains a large fraction of the sphingolipid SM. These two groups of lipids are distinguished by the linker beads connecting their headgroup to the lipid tails. While each of the two linker beads of the glycerophospholipids represents a nonpolar ester group, in the case of SM, one linker bead represents a hydroxyl group and the other an amide group, both of which are polar. Similar to the PC distribution (Fig 1A), SM (Fig 1C) is depleted immediately next to the protein except at TM1, TM5, and TM6, where the sidechains of several polar and charged residues form strong Lennard-Jones interactions with the polar linker beads of SM. Finally, the GM lipids (S2E Fig) are sphingolipids which have an oligosaccharide and sialic acid head group and comprise ~5% of the upper leaflet. During the initial portion of the membrane equilibration the GMs diffuse to the proteins where they remain, very rarely moving back into the bulk membrane. Determining the precise protein-GM interaction, which dictates their segregation propensity, is difficult due to the large headgroup of these lipid species, but it does not appear to be different depending on the receptor conformation. The GMs tend to cluster together in line with the observations of Ingólfsson *et al*. [27] and Gu *et al*. [37].

Composing about 30% of the idealized plasma membrane (S3 Fig), CHOL is one of its largest components. Fig 2 shows the distribution of CHOL around the simulated inactive and active MOR protomers. In agreement with experimental evidence [38], CHOL flip-flops between the upper and lower leaflets and was frequently found to be interacting with the helical bundle region of the receptors, as shown by the structures (Fig 2) colored white to blue to green by the probability of each residue being in contact with the ROH bead of CHOL. For inactive MOR, the highest density of CHOL was found near TM6 and TM7 in the hydrophobic region of the membrane. Notably, an identified CHOL hotspot near TM6 corresponds to the location of the electron density that was attributed to a CHOL molecule in the MOR inactive crystal structure (PDB ID: 4DKL). While a hotspot near the palmitoylation site C170^3.55^ was also observed in the simulations of the inactive MOR, in those of the active MOR, the CHOL was preferentially found between TM2 and TM5 and between TM5 and TM6. Notably, the maximum difference between the CHOL distributions around inactive and active receptors was 0.003 while the maximum standard deviation of CHOL distribution at each grid point of the individual simulation runs was 0.0006, indicating that the differences are substantial.

### Lipid Order and Disorder

**Fig 3:**
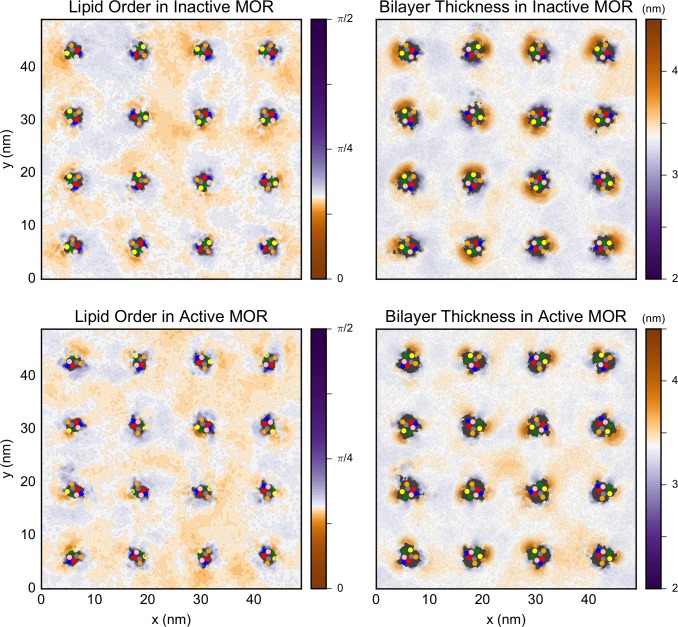
Plots showing the average lipid order (left column) and bilayer thickness (nm, right column) during the final 2 μs of a simulation run of inactive (top row) or active (bottom row) receptors in the low receptor density membrane with position restraints on the receptors. The data is averaged over all lipids, excluding the ones that flip-flop across the membrane (i.e. CHOL, CER, and DAG). For the order, a value of 0 (orange color) indicates a fully ordered lipid tail, and the larger the value, the more disordered the tail. The units of thickness are nm. In both cases, the white color is set to the average value of the simulations with the inactive receptors. The center of mass of the seven TM helices are indicated by the colored dots as follows: TMs 1 through 7 are colored in blue, red, grey, orange, yellow, green, and pink, respectively.

We quantified the local properties of the lipids in the plasma membrane model by calculating the order parameter of each non-flipping lipid species (i.e. excluding CHOL, CER, and DAG) during the final 2 μs of membrane equilibration in which the receptors are kept fixed or the production runs in which the receptors are permitted to freely move in both the high and low receptor density simulations. The lipid order parameter was defined as the angle between the membrane normal and a vector between the first and last bead of the tail. Specifically, as described in the Methods section, a value of the order parameter of 0 means that lipid tails are on average ordered and parallel to the membrane normal, while larger values imply that the lipid tails assume disordered conformations. The extreme value of π/2 means the lipid tail forms a 90° angle with the membrane normal. In Fig 3 and S4 Fig we report, as an example, the results of one of the simulation runs where the active or inactive receptors are fixed in the low or high receptor density systems, respectively. The other trajectories show a similar behavior, as do the trajectories of the mixed-arrays (i.e., 50% inactive/50% active MOR; S5 Fig), which were only simulated in the high receptor density membrane. Since the pattern of ordered and disordered regions near the inactive or active receptors in the mixed-arrays is similar to that in the arrays of only inactive or active protomers, respectively, only the results for all inactive or all active arrays are discussed. There are clear regions of order and disorder in the membrane. As expected, lipid order is correlated with bilayer thickness (see Fig 3, S4 Fig, S5 Fig and S6 Fig), since a lipid with a fully extended tail has a greater z-projection of the head to tail distance than that of a disordered lipid.

**Fig 4:**
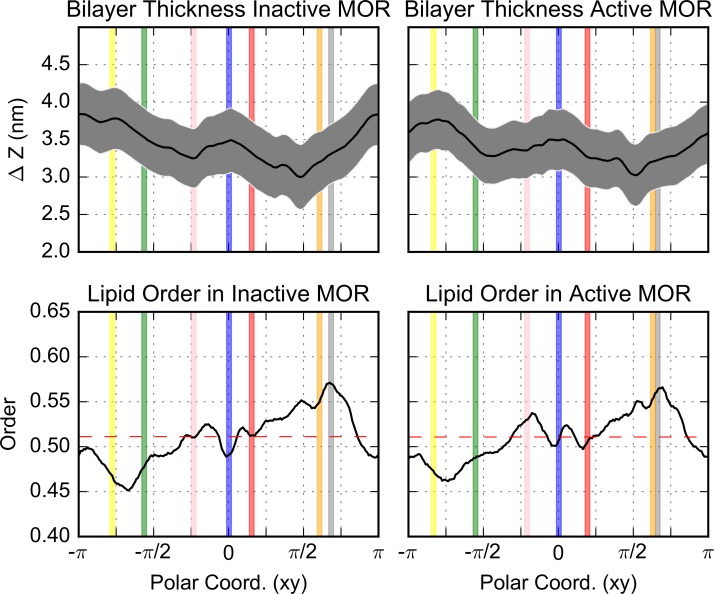
The bilayer thickness (nm, top row) and lipid order (bottom row) of the non-flipping lipids within 3.125 nm of the protein center of mass as a function of the helix index for the low receptor density simulations in which the proteins were permitted to move freely. For the thickness, the average and standard error are indicated by a black line and a grey band, respectively. The overall average value of the order is indicated by a red dashed lines in the bottom panels. The location of the center of mass of the helices is indicated by the vertical lines and TMs 1 through 7 are colored in blue, red, grey, orange, yellow, green, and pink, respectively. Because of the large tilt of TM3, its center of mass appears to the right of TM4.

Fig 3 suggested that the regions close to TM5, TM6, and–to a lesser extent–TM1, show more order while the regions near TM4 show more disorder. To confirm this behavior, we calculated the average membrane thickness and lipid order near the individual helices (shown in top and lower panels, respectively, of Fig 4 for the low receptor density simulations in which the receptor was permitted to move freely). Interestingly, the extent of the order is not only helix dependent, but also conformation dependent (Fig 4). The region near TM5 and TM6 (yellow and green dots in Fig 3, yellow and green lines in Fig 4) is thicker (and more ordered) in the simulations with the inactive receptor than those with active receptors, while the region near TM4 (orange in Figs 3 and 4) is more disordered next to both the active and inactive protomers. The trends in the helix-dependent lipid order are not influenced by the overall protein density nor by the restraints imposed during the equilibration. In fact, similar results were obtained for the low receptor density simulations in which the proteins were permitted to move freely (Fig 4) as well as the low and high receptor density simulations of restrained receptors (S7 Fig and S8 Fig, respectively) despite the inability of the receptors to tilt when they were kept fixed (S9 Fig). The correlation between the locations of ordered regions with SM enrichment was confirmed by calculating a 55% to 45% ratio for the probability to find a SM molecule in a region with order parameter larger or smaller than the average in the high receptor density simulations. For all other lipid species, including CHOL, the two probabilities are equivalent. Notably, the presence of inactive receptors shifts the overall distribution of the order parameter towards smaller values (i.e., higher order) with respect to the active protomers (S10 Fig).

Snapshots of the production runs are shown in S11 Fig for representative high receptor density simulations of inactive and active MOR (upper and lower panels, respectively), and the first 10 μs of a representative inactive trajectory is shown in S1 Movie. All high receptor density simulations show a similar behavior. The lipids closest to the proteins are those that show the broadest distribution of angles. In contrast, in the regions far from the protein, the distribution of order values is much narrower. Thus, the introduction of proteins into a membrane promotes the formation of lipid regions that are either more ordered or more disordered than in the membrane simulated alone. When two receptors get closer together, the lipid regions between them become disordered for the interface to form.

**Fig 5:**
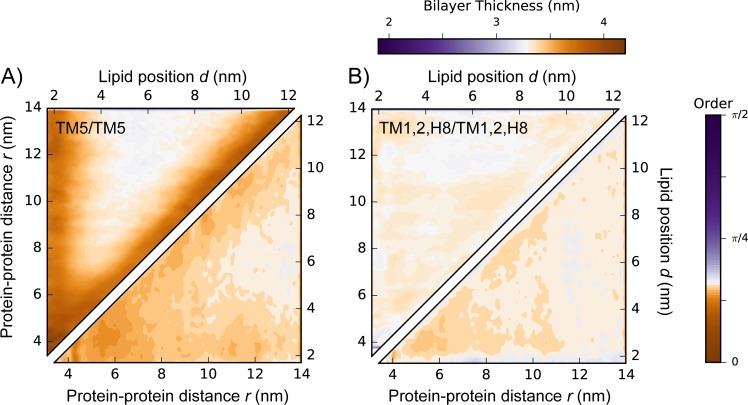
Average lipid thickness (upper triangle) and order (lower triangle) as a function of protein-protein distance *r* and lipid position *d* for interfaces TM5/TM5 and TM1,2,H8/TM1,2,H8 between inactive receptors (panels A and B, respectively).

We then investigated the interplay between the relative lateral position of protomers and the profile of membrane properties, by calculating, for selected interfaces (Fig 5 and S12 Fig), the average order and thickness of the lipids in the region separating two receptors as a function of the distance from the protein center *d* and of the relative protein-protein distance *r*. Not surprisingly, in agreement with the distinct effect of different helices reported above (Fig 4), the membrane profile is also strikingly dependent on the relative orientation of the proteins.

We show this for the two protein regions that are maximally involved in the observed dimers, i.e. TM1,2,H8 and TM5 (see below). Despite both these protein regions favoring the formation of ordered lipid domains, the dependence of such effect on distance is, interestingly, different. In the TM5/TM5 case (Fig 5A and S12A Fig, for the inactive and active receptors, respectively), for well-separated protomers (*r*≫*r*_0_, where *r*_0_ is protomer average radius), the membrane is perturbed up to *d*≈2.5 nm from the protein center, confirming that isolated protomers are accompanied in this case by a stable region of ordered lipids and a thicker membrane. A second regime is established when the protomers are within *r*≈7 nm of each other and cooperative effects of the nearby protomers start to appear. Thus, each protomer appears to start experiencing the influence of the other one at a distance of ~7 nm, which is larger than the sum of the range of the perturbations for the isolated protomers (~2.5×2 ≈ 5 nm). In contrast, for the TM1,2,H8/ TM1,2,H8 interface, the modulation of the membrane properties when protomers are far from each other (*r*≫*r*_*0*_) is very weak on average (Fig 5B and S12B Fig, for the inactive and active receptors, respectively), and the lipid ordering arises almost exclusively from cooperative effects when two protomers are closer than r≈6 nm. The results obtained for asymmetric interfaces, e.g., the TM1,2,H8/TM5 interface (S12 Fig Panels C and D for the simulated systems with inactive or active receptors, respectively) are similar to those obtained for symmetric interfaces, although the recorded cooperative effect is weaker for r<6 nm.

**Fig 6:**
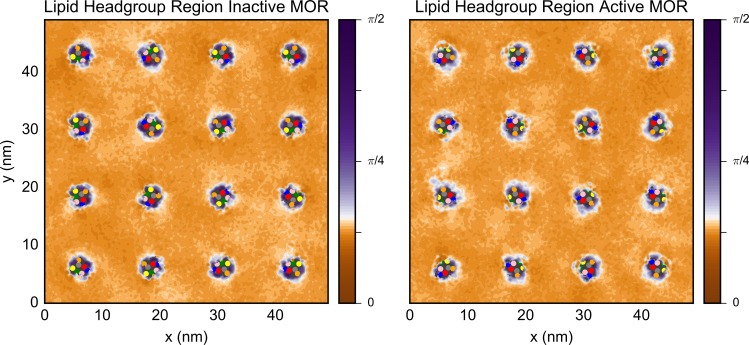
The average order of CHOL molecules in the lipid headgroup region of the upper and lower leaflets next to the inactive receptors (top) and activated receptors (bottom) during the final 2 μs of the low receptor density simulations with the backbone beads kept fixed. In both cases, the white color is set to the average value of the simulations with the inactive receptors. The center of mass of the seven TM helices are indicated by the colored dots as follows: TMs 1 through 7 are colored in blue, red, grey, orange, yellow, green, and pink.

Since CHOL is a key player in the stabilization of ordered phases in plasma membranes [13] and it flips between leaflets, we investigated its ordering behavior separately. While CHOL does not have a tail, it is possible to calculate an order parameter (see Methods section for details) which reveals the orientation of the molecule with respect to the membrane normal (Fig 6). Since CHOL was seen to flip-flop between the leaflets in all simulations, its order was calculated separately for the headgroup regions and the hydrophobic core of the bilayer. Most of the CHOL molecules in the headgroup region were found to be in ordered regions (orange in Fig 6), i.e. they were parallel to the membrane normal, but there were regions close to the protein in which CHOL was tilted (purple in Fig 6), mostly between TM4 and TM5 (orange and yellow dots, respectively). The regions in which the CHOL molecules were tilted roughly correspond to the regions in which the remaining lipids were also disordered (Figs 3 and 4). On the other hand, CHOL molecules away from the protein were, on average, always ordered (i.e., close to parallel to the membrane normal), even when the other lipid regions were disordered. In contrast, CHOL molecules in the hydrophobic part of the membrane exhibited a much broader range of order values (see S13 Fig), which are skewed toward 90°.

### Lipid Residence Time and Transverse Movement

Niemelä *et al*. used CG simulations to show that the mobility of lipids is reduced when they are in the vicinity of proteins [39]. To assess the mobility of lipids near MOR in the high receptor density simulations with freely moving receptors, local residence times were calculated for the lipids with the largest mole fraction (i.e., CHOL and the PC lipids; see S3 Fig) in the proximity of each helix (S14 Fig). Consistent with their homogeneous distribution around the proteins, the residence time of the PC lipids is similar for all the helices (~3.5 ns on average) of both inactive (blue points in S14 Fig) and active (red points in S14 Fig) MOR.

In contrast, the residence time of CHOL differs depending on the helix to which the molecule is in proximity. The CHOL molecules nearest to TM6 stay close to these helices for longer (~5–6 ns for inactive and active MOR, respectively) than the CHOL molecules near the other helices (~4 ns, on average). The CHOL residence time at TM6 or TM7 is longer for the inactive receptor than the active receptor, possibly due to the outward swing of TM6 upon activation.

The only lipids that moved transversely through the bilayer in our simulations, i.e. flip-flopped between the leaflets, were CHOL, CER, and DAG, which are the same lipids that were seen to flip-flop in the published simulations of the plasma membrane without proteins [27]. The rate of CHOL flipping in our low receptor density membrane (inactive: 7.23±0.07×10^6^ s^-1^, active: 7.29±0.03×10^6^ s^-1^) was comparable to the rate of 6.53±0.01×10^6^ s^-1^ in the plasma membrane without proteins [27] as was the rate of DAG flipping (inactive: 6.34±0.30×10^6^ s^-1^, active: 6.62±0.21×10^6^ s^-1^, plasma membrane: 5.87±0.05×10^6^ s^-1^). The flipping rates of CHOL (inactive: 3.97±0.04×10^6^ s^-1^, active: 4.03±0.04×10^6^ s^-1^) and DAG (inactive: 3.38±0.39×10^6^ s^-1^, active: 3.37±0.46×10^6^ s^-1^) in the high receptor density membrane were much slower than in the low receptor density membrane and plasma membrane without proteins. Consistent with the very low rate of flipping seen by Ingólfsson *et al*. [27], the CER switched leaflets very infrequently, on the time scale of our simulations.

**Fig 7:**
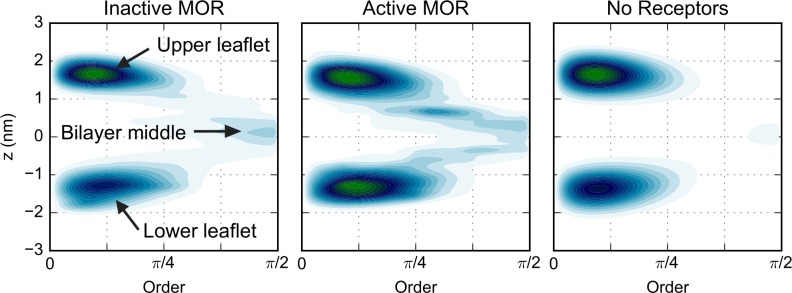
Distribution of CHOL in the plasma membrane with inactive receptors, activated receptors, or no proteins as a function of the z-coordinate and lipid order. The simulations with the receptors are those with high receptor density and the receptor backbone beads kept fixed. The middle of the membrane is set to *z* = 0.

To determine the effect of the protein on the equilibrium distribution of these lipids, we used the final 2 μs of the membrane equilibration of the high receptor density simulations to calculate the density of molecules as a function of the z-coordinate of CHOL’s ROH or CER/DAG’s linker beads (AM1 or GL1) and either (a) the minimum distance to the backbone (BB) beads of inactive or active MOR (S15 Fig) or (b) the lipid order in the plasma membrane with embedded inactive MOR, embedded active MOR, or no receptors (Fig 7 for CHOL and S16 Fig for DAG and CER). The majority of CHOL molecules were found close to parallel to the membrane normal in both the upper and lower leaflets of the plasma membrane with or without receptors. As also seen in S13 Fig, Fig 7 shows CHOL molecules in the hydrophobic region of the membrane that are perpendicular to the membrane normal. Notably, there are more of these perpendicular CHOL molecules in simulations of the plasma membrane with receptors than without them. To confirm that the CHOL distributions are substantially different near the inactive or active receptors, we repeated the analysis for each individual run and found a similar CHOL density in the hydrophobic part of the membrane for all of them. While the concentration of CHOL in the upper and lower leaflets is asymmetric in the simulations of both the inactive receptors and the plasma membrane without receptors, the distribution of these molecules is symmetric in the case of the active receptors. Lastly, an additional distribution of CHOL at an angle between π/3 and π/4 with respect to the membrane normal is seen only in the simulations of the active receptors.

The calculated z-coordinate of CHOL’s ROH beads as a function of their minimum distance from the protein’s BB beads (S15 Fig) shows minima in the middle of the bilayer (-0.8 nm < z < 0.8 nm) that are immediately next to the protein (minimum distance ~0.5 nm), suggesting that the protein binds CHOL at these sites, consistent with the regions of high cholesterol density seen in Fig 2. A kinetic model was built to determine whether these minima in the hydrophobic region of the bilayer next to the protein are involved in the flipping mechanism of CHOL. The five most frequently occurring pathways of CHOL movement in the z-direction are shown in S17 Fig. The two largest states (6 and 7 in S17 Fig) in the models for both the active and inactive simulations correspond to CHOL in the upper or lower leaflets, respectively, which have no contacts with the protein. For both the inactive and active protomers, the largest fluxes were between states 6 and 7 indicating that the main route of CHOL flipping is through the membrane away from the proteins. The most probable pathway for a CHOL molecule from the upper to lower leaflet through a bound state is via state 1 for the inactive protomer and state 4 for the active protomer, although the flux is much lower through this pathway than through the membrane.

The distributions of CER and DAG lipids were also examined, although these lipids each make up less than 1% of the membrane. The location of the deepest minimum in the plots of the order of DAG lipids as a function of z are different in the simulations of the plasma membrane with or without receptors (S16 Fig). For the inactive receptors, the deepest minimum of DAG lipids is in the middle of the bilayer, while it is in the lower or upper leaflet for the active receptors or membrane without proteins, respectively. In contrast, the distributions of the CER lipids are similar among themselves, except for a shallow minimum in the middle of the bilayer in the case of the simulations of the inactive receptors.

### Interfaces Formed by Inactive/Inactive, Active/Active, or Inactive/Active Receptors

Removing the position restraints on the receptor BB beads allowed the receptors to move freely in the membrane and to eventually self-assemble within the initial microseconds of the high receptor density simulations. While a quantitative assessment of the dimer formation kinetics cannot be obtained with our data, the time decay of the number of monomers in the membrane (S18 Fig) shows that the inactive system forms aggregates slightly more quickly than the active. However, once formed, the interfaces did not dissociate over the 20 μs of simulation time.

**Fig 8:**
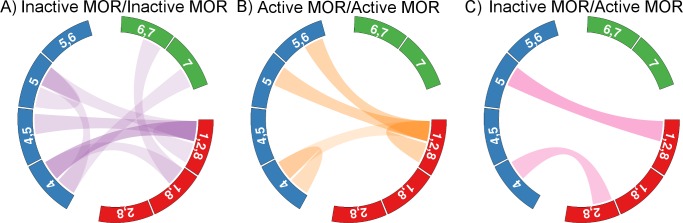
Schematic of the interfaces formed by a) two inactive receptors, b) two active receptors, and c) one active and one inactive receptor. The opacity of the color linking the two sides indicates the probability of formation

To characterize the structural features of the formed complexes, the interfaces formed by the final microsecond of simulation time were clustered by their contact maps. While the simulations do not provide enough statistics to definitively quantify the stability of each interface, their formation or absence is telling. The fraction of interfaces formed is listed in S2 Table and depicted in Fig 8A, 8B and 8C for dimers formed between two inactive receptors, two active receptors, or one active and one inactive receptor, respectively.

There is only one interface that was formed by inactive/inactive, active/active, or inactive/active receptors, i.e. irrespectively of the conformation of the participating protomers: TM1,2,H8/TM5. In this interface, TM2 only forms extracellular contacts with TM5, while H8 and TM5 form intracellular contacts.

For the inactive proteins, nine different interfaces were formed, but over 50% of them involved the TM1,2,H8 region of one protomer and the TM4/TM5 region of the other. In particular, the TM1,2,H8/TM4,5 interface constituted the ~15% of the observed interfaces while the similar interfaces TM1,2,H8/TM4 and TM1,2,H8/TM5 made up ~31% and 8%, respectively, of all observed interfaces. In the TM1,2,H8/TM4 and TM1,2,H8/TM5 interfaces, the second protomer is slightly rotated either clock- or counter-clockwise relative to that of the TM1,2,H8/TM4,5 interface, such that no contacts are formed with TM5 and TM4, respectively. In all of these interfaces, TM2 forms extracellular contacts with TM4,5, while the majority of the contacts are formed by TM1. The inactive receptors formed two interfaces involving TM7, which were not observed in the active or mixed proteins. In the TM4/TM7 (~7%) interface, TM7 is in contact with TM4 on the intracellular side, while TM7 forms extracellular contacts with TM1 in the TM1,H8/TM6,7 interface (~8%). The TM1,2,H8/TM1,2,H8 (~8%), one of the crystallographic interfaces, TM4/TM5 (~8%) and TM5/TM5 (~8%) interfaces were also observed in the simulations of inactive MOR. While the TM5/TM5 interface met our criteria for interface formation (see Methods section), it is not as compact as the other interfaces with some lipid tails located in between the protomers.

The most frequently formed interfaces in the simulation of all active MORs are the TM1,2,H8/TM5,6, TM1,2,H8/TM1,2,H8, and TM1,2,H8/TM5 interfaces which are all seen with approximately the same frequency (~25%). In the TM1,2,H8/TM5,6 interface, TM6 forms contacts on the extracellular side only, which allows the interface to form without preventing the outward swing of TM6 in the active structure. The contacts formed by TM1 and TM2 in the TM1,2,H8/TM1,2,H8 interface are on the extracellular side, while H8 forms contacts on the intracellular side. Another frequently seen interface between active receptors is the TM4/TM4 interface (~17%) in which contacts are formed only by TM4. Both the TM4/TM4 and TM1,2,H8/TM5,6 interfaces were unique to the active protomers.

In the case of the dimers in which one protomer was in the inactive conformation and one was in the active conformation, only two interfaces were formed. However, what appeared as a new interface, TM2,H8/TM4, was structurally very similar to the TM1,2,8/TM4 interface formed by inactive receptors except that one protomer was slightly rotated, lengthening the distances between TM1 and TM4 beyond our threshold to form a contact. The TM1,2,H8/TM5 asymmetric interface is the most favored (~60%) and is formed by either an inactive/active or active/inactive combination. The symmetric TM1,2,H8/TM1,2,H8 interface was not formed by the combination of one active and one inactive protomer, which is surprising since it was formed by either two inactive protomers or two active protomers. In the crystal structures of the two conformations, the intracellular ends of TM7 do not overlap, shifting H8 slightly. Thus, formation of the TM1,2,H8/TM1,2,H8 interface in this mixed system may require some adjustments in the position of H8, which the model is not able to capture due to the elastic network required to maintain the receptor tertiary structure.

The highest-order oligomer seen in the simulations was a trimer, but only few of them were recorded. For instance, only three trimers were seen in the simulations of inactive receptors with the following interfaces: 1) TM7_A_/TM4_B_ and TM4_A_/TM1,2,H8_C_, 2) TM5_A_ /TM5_B_ and TM4_A_ /TM5_C_, and 3) TM4_A_/TM1,H8_B_ and TM1,2,H8_A_/TM4_C_, where subscripts A, B, and C identify the three protomers participating in the trimer. Only one trimer was seen in simulations of all active receptors, and it consisted of a TM1,2,H8_A_/TM1,2,H8_B_ interface and a TM5,6_A_/TM1,2,H8_C_ interface. In the mixed-array simulations, one trimer formed as the result of the association of two active receptors (B and C) with an inactive receptor (A) in the middle. In this configuration, the inactive protomer contributed TM5 to a TM5_A_/TM1,8_B_ interface and TM2,H8 to a TM2,H8_A_/TM4_C_ interface.

### Lipid Distributions around Dimers

The distribution of the lipids around frequently occurring interfaces in the inactive (e.g., TM1,2,H8/TM4 and TM1,2,H8/TM4,5) and active (e.g.,TM1,2,H8/TM5,6, TM1,2,H8/TM1,2,H8, or TM1,2,H8/TM5) dimers was calculated to determine possible correlations between the location of lipids near the protomers and the formation of specific dimeric interfaces. The concentration of the PA, PS, PI, and PIP_1-3_ lipids in the lower leaflet is too low to draw robust conclusions on any role these lipids play at the interface. While the distributions of the PC, PE, and SM lipids revealed no specific hotspots at dimer interfaces, CHOL molecules were always present at the dimer interfaces for the five interfaces listed above. The model structures in S19 Fig, which are colored according to the probability of a CHOL molecule being in contact with the helices involved in a dimer interface, provide an example of these interactions at the TM1,2,H8/TM4 and TM1,2,H8/TM1,2,H8 interfaces formed between inactive or active receptors, respectively and the residues frequently in contact with the bundle are listed in S3 Table.

All of the residues with which CHOL forms contacts for more than 50% of the simulation are on the helical bundle. As seen in S3 Table, four out of five interfaces involving TM1 exhibit contacts of CHOL with A73^1.37^, S76^1.40^, I77^1.41^, and T120^2.56^. All five interfaces exhibit CHOL contacts with L116^2.52^ and S119^2.55^. While S119^2.55^ has one of the highest probabilities of being in contact with CHOL when MOR is monomeric, I242^5.48^ is frequently in contact with CHOL in both protomeric and dimeric configurations. The three interfaces involving TM5 have CHOL most frequently bound at I238^5.44^, F241^5.47^, I242^5.48^, M243^5.49^, and V245^5.51^.

## Discussion

### Lipid Order/Disorder Facilitates Dimerization

Studies from several groups [6,19,40–43], including ourselves [28,44], have computationally explored the effects of the lipid bilayer on the formation of GPCR complexes. In this work, we further investigate the impact of the lipid environment on the spatio-temporal organization of inactive and/or active MOR employing, for the first time, a more realistic 63-component plasma membrane [27] model.

The effects of lipids on protein association in the membrane are classified as either specific (i.e., direct) or non-specific effects [45]. Specific effects refer to possible interactions of individual lipid molecules with specific residues on the protein surface, whereas non-specific effects are attributed to the modulation of the properties of the membrane (e.g. bilayer thickness). Our analysis of MOR simulations shows an interesting interplay between these two types of effects with individual helices of the receptor promoting regions of the membrane with different average order. Specifically, the enrichment of SM at helices TM1, 5 and 6 promotes regions of ordered lipid molecules next to these helices, while helix TM4 is most frequently adjacent to less ordered regions of the lipid.

Recently, Katira *et al*. [46] suggested a mechanism by which the stabilization of phases with specific order by proteins in a homogeneous membrane can lead to membrane-mediated interactions between proteins. In their model, a disordered phase of a simple DPPC membrane was stabilized around an idealized protein by setting the length of the protein hydrophobic core to be less than the thickness of the surrounding ordered membrane. When two proteins, each surrounded by their own disorder lipid phase, approach each other, it is energetically favorable for the two regions of disordered lipids to merge to minimize the size of the order/disorder interface, resulting in an effective, long-range, induced interaction between the proteins. A similar mechanism appears to be occurring in the MOR simulations reported here. However, in contrast to the homogeneous idealized proteins studied by Katira *et al*., MOR in the heterogeneous membrane appears to induce order/disorder depending on the helix and the lipids closest to it. In the complex plasma membrane in our simulations, the hydrophobic length of the individual helices as well as the modulation of the local bilayer compositions by the protein produce specific hydrophobic mismatches that lead to helix-dependent regions of order and disorder. Despite these important differences, the simulations reported here show how ordered lipid regions will tend to coalesce to reduce the energetic cost of merging ordered and disordered regions in the membrane.

Although the high-protein concentration in our high receptor density simulations, and the limited length of the low receptor density simulations prevent us from addressing the effect of this mechanism on the translational dynamics of the proteins since the average protein-protein distance is relatively small in our system, it is clear that the lipid order influences the orientation of protomers that eventually come together to dimerize. Specifically, interfaces formed by helices next to order-inducing regions (e.g. TM1,2,H8/TM5) are more frequently formed than those next to disorder-inducing regions (e.g. TM4/TM4) or those next to regions with opposite order preference. At a shorter range, the proteins will then proceed to form bona fide dimeric structures depending on specific residues at the interface, shape complementarity, and physico-chemical properties of the interface. The presence of ordered lipid regions next to helices TM1, 5, and 6 fades when the distance between protomers is only a few nm. On the nanometer length scale it is reasonable to assume that shape complementarity and specific interactions start playing a role in dictating the shape of the interaction free-energy and ultimately determining whether an interface can form or not. Thus, whether the helices involved in interface formation induce ordered or disordered lipid regions ceases to play a role once the protomers are close together.

Our simulations show an area of high CHOL density near the palmitoylated C170^3.55^ located between TM4 and TM5 of MOR, which had been previously suggested to trap CHOL molecules and to promote dimerization [5]. This palmitoylation site appears to help order the lipids around TM5 in simulations of the inactive but not the active MOR which may explain why the TM5/TM5 interface is formed between inactive but not active receptors. While increasing the lipid order and the membrane thickness near TM4 enhances the propensity of inactive MOR to form a dimerization interface involving this helix, the outward movement of TM6 upon activation appears to affect the ability of this helix to be involved in dimerization of active MOR by virtue of a decreased lipid order and membrane thickness near that helix.

The results presented here offer testable hypotheses for experimental investigation. For instance, regions of lipid order immediately next to the MORs are found to be rich in SM, which is known to contribute to ordered regions of a membrane [47]. We find that the polar linker beads of SM lipids associate with polar/charged side chains on TM 5 and 7 of MOR (e.g. Y227^5.33^, N230^5.36^, F313^7.30^, Q314^7.31^). Mutating these residues to Ala might decrease lipid order and interface formation since the attraction between SM and the protein is predicted to decrease.

Lipid rafts have long been thought to compartmentalize cellular processes by contributing to the assembly of signaling molecules [48]. There is support for localization of activated MOR in lipid rafts [32,35], but the involvement of lipid rafts in GPCR signaling is likely dependent on the signaling pathway and the cell type [49]. The plasma membrane used in the simulations reported herein is too small to eventually see formation of lipid rafts, but we can speculate that the ordered/disordered lipid regions mimic the role of lipid rafts in guiding the assembly of receptors, and may also contribute to orienting the receptors so as to guide their interaction with specific regions of the intracellular proteins, e.g., the G-proteins, that are in contact with the membrane.

### Direct Lipid-Receptor Interactions Do Not Trigger Transbilayer (Flip-Flop) Lipid Motion

Transmembrane lipid translocation, or flip-flop, is essential to maintain the required composition of cell membranes. In the simulation reported here, only three lipid types were observed to flip-flop from one leaflet to the other, specifically CHOL, CER, and DAG, which are the same three lipids found to flip-flop in published simulations of the plasma membrane without proteins [27]. Although GPCRs have recently been shown to act as phospholipid scramblases [50], helping them move from one leaflet to the other, phospholipids did not flip within the 20 μs of our simulations. This is likely due to the timescale of our simulations. Notably, published umbrella sampling simulations of different sets of lipid bilayers showed that the energy barrier for a dipalmitoylphosphatidylcholine (DPPC) lipid to flip-flop though a DPPC bilayer is increased by 26 kJ/mol for a 20% CHOL membrane relative to a pure DPPC bilayer [51]. The flipping rate of DPPC was estimated to be on the order of hours in a pure membrane and on the order of years in a DPPC/CHOL membrane.

The distribution of CHOL in the hydrophobic region of the bilayer (S13 Fig, solid lines) show that the CHOL molecules are either perpendicular to the membrane normal or close to perpendicular, which is consistent with neutron scattering experiments showing the presence of disordered CHOL in a PC membrane with polyunsaturated tails [52]. Previous simulations have shown that CHOL flips in a membrane by first tilting, and then migrating to the middle of the bilayer where it is perpendicular with respect to the membrane normal [53–55]. While we also see CHOL in the middle of the membrane where it is bound to the protein, our kinetic model shows that the main route for CHOL flipping is through the membrane away from the proteins. The mechanism by which transmembrane proteins promote lipid flip-flopping has been attributed to either thinning of the membrane near the protein or the formation of hydrophilic interactions between lipid and protein, both of which would reduce the energy barrier to flipping. Here we see that CHOL flipping via states that are bound to the protein reduces the overall translocation rate, suggesting that the same would be true for other lipids with hydrophilic heads.

The dimer structures also showed CHOL bound to the helical bundle. The helices to which CHOL binds when MOR is isolated are not the same helices to which it binds when the receptor is in a dimeric configuration. However, there is no correlation between CHOL bound at specific regions of the helical bundle and dimerization. It is unclear if the CHOL molecules bind an individual protomer before the interface forms or if they insert themselves between protomers after dimerization. The limited statistics of the simulations presented here does not allow us to derive quantitative inferences about the behavior of individual CHOL molecules at the dimer. A new set of simulations is currently being performed to answer this question.

### Lipids with Negatively Charged Headgroups Appear to Play a Role in Activation but not Oligomerization

The non-sterol lipids which make up the bulk of the membrane (PC, PE, SM; 60% in the upper leaflet and 53% in the lower leaflet) show a ring-like distribution around the individual receptor protomers. While some of these zwitterionic lipids are localized around the helices, these preferences are mostly independent of the protein conformation. In contrast, the negatively charged PIP lipids which are present only in the lower leaflet have significant differences in their distribution around the inactive and active conformations supporting experimental inferences that they favor the latter conformation. The PA, PS, and PI lipids all have a charge of -1, but the PIP_1-3_ lipids are derivatives of the PI lipids which have been phosphorylated once (PIP_1_), twice (PIP_2_), or three times (PIP_3_) and thus have charges of -3, -5, and -7 respectively. These lipid distributions are consistent with experimental and computational evidence showing that negatively charged residues promote activation not only in GPCRs [10,11,56] but also in ion channels and transporters [57]. For instance, in the case of β_2_AR, the presence of the negatively charged lipid phosphatidylglycerol (PG) promoted agonist binding and activation while the neutral PE lipid promoted antagonist binding and the inactive conformation [10]. Recent all-atom MD simulations of β_2_AR showed that a PG lipid can insert itself inside the receptor between TM6 and TM7, forming a salt bridge with R3.50 and stabilizing active conformations [56]. While the rigidity of the elastic network applied to the coarse-grained receptor structure in our simulations prevents lipids from entering the MOR, the PIP_1-3_ and PA lipids prefer to localize at the crevice between TM6 and TM7 formed upon activation of the receptor as a result of TM6 swinging outward. The ability of negatively charged lipids to promote activation has also been shown for another GPCR, the neurotensin receptor NTS1, as coupling between NTS1 and Gq-proteins increased with PG content [11]. The results of our simulations are consistent with a conformation-dependent role of negatively charged lipids in membrane protein function, but due to the low concentration of these lipids in the idealized plasma membrane, we cannot derive a clear conclusion about their role, if any, in oligomerization.

### Interface Formation Is Affected by Receptor Conformation

Early cysteine cross-linking experiments have suggested that inactive and active GPCRs do form different interfaces [58–60]. The present simulation results show that the interfaces formed by the MOR receptors are also dependent on the conformation of the protein. Specifically, while both the inactive and active receptors formed the TM1,2,H8/TM1,2,H8, TM1,2,H8/TM4, and the TM1,2,H8/TM5 interfaces, each conformation also formed mutually exclusive interfaces.

Both the inactive and active crystal structures of MOR show dimeric packing interactions involving TM1, TM2, and H8. The Cα RMSD of TM1 and TM2 of the simulated inactive/inactive dimer was 4.2 Å (referenced to the MOR inactive crystal structure 4DKL) or 2.8 Å (referenced to the MOR active crystal structure 5C1M). In the case of the active/active simulated dimer, the RMSD was 4.8 Å (referenced to 4DKL) or 2.9 Å (referenced to 5C1M). Unlike the crystal structure of active MOR, the crystal structure of inactive MOR also showed a symmetric TM5,6/TM5,6 packing interaction [29]. The fact that this interface is neither seen here nor in previous simulations we carried out on the inactive MOR in a POPC/10% CHOL environment [28] further supports the suggestion that the TM5,6/TM5,6 interface is either kinetically impaired or it may represent a crystallographic artifact.

Similar to the results of our previous simulations of inactive MOR in a POPC/CHOL membrane [28], the simulations carried out here suggest that interfaces TM1,2,H8/TM1,2,H8, TM1,2,H8/TM4,5, and TM5/TM5 are likely to form in a mimetic membrane environment. In contrast, the TM1,2,H8/TM5,6 and TM4,5/TM5,6 interfaces seen in the POPC/CHOL membrane did not form between inactive protomers in the 63-component plasma membrane, but we believe that this is most likely due to differences in the modeled intracellular loop 3 (IL3).

Several other GPCR crystal structures show packing interactions that are similar to those seen in our simulations. Both the inactive δ-OR (DOR, PDB ID: 4DJH) [61] and inactive β_1_ adrenergic receptor (β_1_AR, PDB ID: 4GPO) [62] crystal structures form TM1,2,H8/TM1,2,H8 interfaces. The Cα RMSD of the inactive/inactive simulated dimer was 3.8 Å relative to DOR and 2.8 Å relative to β_1_AR. Two of the asymmetric interfaces resulting from the simulations of inactive MOR were seen in the crystal packing of chemokine receptors: TM1,H8/TM6,7 in CXCR4 (PDB ID: 3OE8) [63] and TM4/TM7 in CCR5 (PDB ID: 4MBS) [64], with RMSD of 4.5 Å and 4.4 Å, respectively. Interestingly, the chemokine receptors have been suggested to form heterodimers with MOR [65]. Another interesting observation is that no higher-order oligomers other than trimers are identified in our simulations, but this may be due to our choice of strict interface criteria, which limits the higher-order complexation to trimers in the afforded timescale.

In summary, we have performed over 300 μs of CG MD simulations of inactive and/or active MOR in an idealized plasma membrane and concluded that the impact MOR conformation has on dimerization is two-fold. First, the two conformations induce different patterns of order and disorder with merging ordered regions determining protomer orientation with respect to one another. Second, the shape complementarity between different conformations affects both the number and type of interfaces formed. While indirect lipid effects are found to play a major role in receptor dimerization, direct effects through specific lipid-receptor interactions require further investigation.

## Methods

### System Set-Up

The inactive and active crystal structures of the mouse MOR (PDB ID: 4DKL [29] and 5C1M [30], respectively) were used as the starting structures after removal of all non-receptor atoms (e.g. the ligands, as well as the fused T4L lysozyme and the nanobody in the inactive and the active crystal structures, respectively). The missing intracellular loop 3 (IL3) of the inactive structure was added using the high-resolution structure of the δ-OR (PDB ID: 4N6H) [61] as a template for homology modeling. The N-terminal region of the active structure was removed, while the missing residues of helix 8 (Η8) were added so that both proteins consisted of residues 65 to 352. All missing residues and side chains were added using MODELLER [66]. The resulting structures were coarse-grained according to the Martini force field version 2.1 [67–69] using the martinize.py script. Receptor tertiary structure was maintained with a modified version of the elastic network [70,71]. Specifically, a harmonic force was applied between all BB beads within a cutoff of 0.9 nm using a force constant of 1000 kJ mol^-1^ nm^-2^ for helical residues, or 250 kJ mol^-1^ nm^-2^ for residues in unstructured regions. In agreement with experimental inferences [5], C170^3.55^ (the superscript follows the Ballesteros-Weinstein numbering scheme [72]) was palmitoylated by adding four C1 beads with a bond length of 0.47 nm and force constant of 1250 kJ mol^-1^ nm^-2^ and angles of 180° with a force constant of 25 kJ mol^-1^ rad^-2^.

Two membrane sizes were used for the simulations reported in this work. Arrays of 16 coarse-grained proteins were placed in either a large membrane patch of 50×50 nm^2^ or a smaller patch of 25×25 nm^2^, corresponding, respectively, to 6.4×10^3^ and 2.5×10^4^ receptors/μm^2^. We use “low receptor density” to refer to the large membrane patch and “high receptor density” to refer to the small membrane patch. The proteins were evenly spaced, and each of the protomers was randomly rotated around its z-axis. Two types of arrays were created, using all inactive or all active receptors, respectively. For each of the four protein set-ups (active and inactive conformations, high and low receptor density), five sets of arrays were created for a total of 20 starting protein configurations. Furthermore, a third type of array, containing 50% inactive/50% active (hereafter indicated as “mixed arrays”) receptors, was prepared (in five replicas) for the high receptor density setup, adding 5 more starting receptor configurations. In the case of the mixed arrays, 50% of the proteins were randomly assigned to be inactive, while the others were in the active conformation (see S20C Fig for an example of an initial configuration of mixed arrays for the high density system, while S20 Fig Panels A and B show examples of all inactive and all active receptor set-ups for the high receptor density systems, respectively).

Using the insane.py [73] script, the protein arrays were then embedded in a coarse-grained 63-component plasma membrane with the same composition as that obtained by Ingólfsson *et al*. [27] (see S3 Fig) scaled so that the protein to lipid ratio was approximately 1:200 and 1:100, respectively for the low and high receptor density setups. The total number of lipids in each membrane was approximately 3200 (or 800) in the upper leaflet and 3000 (or 750) in the lower leaflet for the low receptor density (or high receptor density) systems. As in Reference [27], a 2 kJ mol^-l^ nm^-2^ restraint was placed on the phosphate bead of POPC and PIPC lipids in the upper leaflet in the z direction to prevent large membrane undulations. The height of the box was set to 11 nm.

The protein-membrane systems were solvated with water, and ions were added to neutralize the total charge. To provide a direct comparison of the plasma membrane without proteins, the final structure from the 40 μs trajectory of Ingólfsson *et al*. [27] was retrieved from the Martini force field website (http://md.chem.rug.nl) and used as the starting point for a 700 ns trajectory using the same settings as the simulations with the receptors.

### Simulation Details

Following 10,000 steps of steepest descent energy minimization, the systems were simulated for 100 ns using a timestep of 10 fs keeping position restraints on the backbone beads of the receptor. To equilibrate the membrane and determine the distribution of the lipids around the individual protomers, each system was run for 5 μs using a timestep of 20 fs, while still maintaining position restraints on the receptors. In preparation for the production run, four 10 ns runs with decreasing restraints on the proteins (500, 100, 50, and 10 kJ mol^-1^ nm^-2^) were performed. The production runs were 20 μs and 3 to 5 μs long for the high receptor density and the low receptor density systems, respectively, giving a cumulative time of over 300 μs for the three simulated protein combinations. The simulations were run in the NPT ensemble, with reference temperature of 310 K controlled with the v-rescale algorithm (τ_t_ = 1.0 ps) [74], and reference pressure of 1 bar, controlled with the Berendsen algorithm (τ_p_ = 5.0 ps) [75]. The Coulomb interactions between 0 and 1.2 nm decayed smoothly to 0, while the van der Waals interactions between 0.9 and 1.2 nm decayed smoothly to 0. All simulations were performed with GROMACS 4.6 [76,77].

### Lipid Analysis

The analysis of the lipids around receptor protomers was performed on the final 2 μs of the membrane equilibration part of the simulation–in which the proteins were kept fixed with position restraints–and on the protomers extracted from the production runs. In the latter, a receptor was considered monomeric if the distance between the center of mass of the protein and every other protein was at least 5 nm. Since the atomic coordinates were recorded every ns and each frame has 16 receptors, each lipid distribution around the receptors was calculated from 5×2000×16 sets of lipid-protein positions. The analysis of the lipids around the dimers was performed on the frames from the final 1 μs of simulation time of the high receptor density production runs, which was also used for interface clustering. The ROH beads of the CHOL or the first linker bead (GL1 or AM1) of the non-sterol lipids were used for the lipid analysis (see S1 Fig for a depiction of representative lipids). Lateral lipid density was calculated by binning the position of the lipid beads into 50×50 square bins with a side of 0.2 nm in the membrane plane and calculating the normalized probability distribution. In order to quantify the order and disorder of the lipid tails, the angle between the average membrane normal (*z* direction) and the vector from the linker bead (AM1/2 or GL1/2) to the last bead of the tail was calculated for both tails of each lipid. While the metric used to calculate the lipid order, which is akin to that suggested by Katira *et al*. [46], does not follow the traditional definition of lipid order parameter, it is more computationally efficient, and it allows us to use the same metric to characterize the order of non-sterol lipids and CHOL. While CHOL does not have a tail, the angle was calculated between the membrane normal and the vector from the ROH bead to the final bead. For CHOL, the membrane was split in three parts with the middle of the bilayer defined to be 1.6 nm thick by plotting the histogram of the *z*-coordinate of the ROH bead. Order distribution plots were obtained by averaging the order in 1.75×1.75 Å^2^ square bins parallel to the *xy* plane. The local thickness of the membrane was defined as the difference between the average *z*-coordinate of the linker bead to which the headgroup is attached (AM1 or GL1) in each of the two leaflets, on the same 1.75×1.75 Å^2^ grid used to calculate the order. The dependence of the membrane modulation on the relative position of proteins was analyzed by selecting all frames in both the high receptor density and low receptor density production runs with two protomers at a given distance *r* = ||**R**_1_–**R**_2_|| (where **R**_1_ and **R**_2_ denote the COMs of the two proteins), and with relative orientation described by angles α and β in the regions α∈Ω_α_ = [α_0_–*π*/6, α_0_+*π*/6] and β∈Ω_β_ = [β_0_–*π*/6, β_0_+*π*/6], where α_0_ and β_0_ specify the region of the two protomers facing the dimerization interface (0 or –3/4*π*, respectively for the TM1,2,H8 and TM5,6, interfaces). The position lipid molecules relative to the protomers was described as *d***v**_**d**_+*n***n**_**d**_ in the frame of reference given by the versor **v**_**d**_∝**R**_1_–**R**_2_ and its normal **n**_**d**_. We report averages of membrane thickness and order over Ω_α_, Ω_β_, and *n<r*_*0*_ (where r_0_ = 1.7 nm is the average protomer radius), as a function of *r* (protomer distance) and *d* (lipid position). Only frames for which no other protein was found within *r*_0_ from the line connecting the COMs (i.e., *n*<*r*_0_) of the two protomers were included in the analysis. The averages are calculated and reported for *d*∈[*r*_0_, *r*–*r*_0_].

A kinetic model of the CHOL movement was generated with PyEmma [78] using the contacts formed between the ROH and BB beads to perform the geometric clustering. A hidden markov model was used to kinetically lump the clusters into 8 macrostates.

The mean residence time of the lipids around a helix of the monomer was calculated for the production runs together with the last 2 μs of their corresponding equilibration runs. For every lipid, the length of time spent within 1.2 nm of any sidechain bead (SC) of each helix was calculated in order to obtain a distribution of residence times per lipid species and helix per run. The total mean residence times and standard deviation were obtained by averaging over the distributions from each replica.

### Interface Analysis

All interface analysis was performed on the high-density simulations. To characterize the interfaces formed during the production runs, k-means clustering was performed on the final 1 μs of the trajectories, using the Euclidean norm d^2^ = Tr(D^T^D) of the difference of contacts maps D as a dissimilarity measure. An interface was considered to be formed when at least ten residues on each protomer formed contacts with the other protomer, with a contact defined as two backbone beads lying within 0.8 nm of each other. Helices with three or more residues forming contacts were used in the interface name. The relative frequency of each observed interface and their variance were estimated using a multinomial model X_j_ ~ Multinomial(n,p_j_), where X_j_ is the number of observed dimers for interface *j* and *n* is the total number of observed dimers. Specifically, the well-known relation to the Poisson model was exploited to rewrite the model as X_j_ ~ Poisson(λ_j_) and p_j_ = λ_j_/∑ λ_j_. We took a Bayesian approach, and sampled the posterior distributions of p_j_ with normal uninformative (standard deviation 100) priors on γ_j_ = log(λ_j_) using the observed values of X_j_ for each trajectory. Results are reported as mean and 95% credible intervals. Sampling was performed with the rstan 2.8.0 interface to the Stan language [79]. The RMSD of the simulated interfaces relative to the crystal structures was determined after aligning the Cα of the two dimers. The RMSD was calculated using only the Cα atoms of the helices participating in the interface to ensure that the RMSD captured only the differences between the interfaces. The structures of the interfaces were rendered in pymol. Scripts for the lipid analysis and interface clustering employed the MDAnalysis python libraries [80].

## Supporting Information

S1 Table The fraction of the final 2 μs of the low receptor density simulation with fixed receptor backbone (BB) beads during which the BB beads of the residues are in contact with the GL1 bead of a PIP_1-3_ lipid. All residues listed are in contact with either the inactive or active receptor at least 10% of the simulation time. Residues exhibiting the greatest difference between the two receptor conformations are shown in bold.
(PDF)

S2 Table Fraction of interfaces formed between inactive, active, and inactive/active MOR determined by clustering the conformations observed during the final microsecond of the high receptor density trajectories in which the receptors were free to move. The mean fraction of dimers belonging to each specific interface is reported along with its 97.5% credible intervals. The most frequently observed interfaces for each dimer type are highlighted in bold.
(PDF)

S3 Table The residues on helices involved in dimer interfaces with which CHOL is in contact for more than 50% of the final microsecond of the high receptor density simulations with the receptors free to move. Residues in contact with CHOL for more than 75% of the simulation time are in bold.
(PDF)

S1 Fig Structure of A) a cholesterol, B) a PC/SM lipid, and C) a GM lipid with the ROH, GL1, and AM1 beads used in the lipid analysis colored in cyan. The headgroup beads are in pink and the remaining tail beads in grey.
(PDF)

S2 Fig Lipid distributions of A) PA, B) PI, C) PIP_1-3_, D) PS, and E) GM around individual protomers during the final 2 μs of the low receptor density simulations with the BB beads of the receptors fixed. The dots indicate the centers of mass of the BB beads of the receptor helices.
(PDF)

S3 Fig The fraction of lipids with each headgroup in the plasma membrane at the start of all of the simulations. ‘Other’ in the upper leaflet includes: CER (0.7%), LPC (1.0%), and DAG (0.4%). ‘Other’ in the lower leaflet includes: PA (1.5%), PIP_1-3_ (1.6%), CER (0.1%), and DAG (0.5%). The total number of lipids in the upper and lower leaflets was approximately 805 and 750, respectively for the high receptor density membrane and 3200 and 3000 for the low receptor density membrane.
(PDF)

S4 Fig Plots showing the average lipid order (left column) and bilayer thickness (nm, right column) during the final 2 μs of membrane equilibration for one high receptor density simulation run of inactive (top row) or active (bottom row) receptors with fixed BB beads. The data is averaged over all lipids, excluding the ones that flip-flop across the membrane (i.e. CHOL, CER, and DAG). For the order, a value of 0 (orange color) indicates a fully ordered lipid tail, and the larger the value, the more disordered the tail. The units of thickness are nm. In both cases, the white color is set to the average value of the simulations with the inactive receptors. The center of mass of the seven TM helices are indicated by the colored dots as follows: TMs 1 through 7 are colored in blue, red, grey, orange, yellow, green, and pink, respectively.
(PDF)

S5 Fig Plots showing the average lipid order (left) and bilayer thickness (right) for one high receptor density simulation run of a mixed array of inactive and active MOR during which time the BB beads of the receptors were fixed. The data is averaged over all lipids, excluding the ones that flip-flop across the membrane (i.e. CHOL, CER, and DAG). For the order, a value of 0 indicates a fully ordered lipid tail, and the larger the value, the more disordered the tail. The units of thickness are nm. In both cases, the white color in the color bar is set to the average value of the simulations with the inactive receptors. The centers of mass of the seven TM helices are indicated by the colored dots as follows: TMs 1 through 7 are colored in blue, red, grey, orange, yellow, green, and pink, respectively. The ‘I’ and ‘A’ indicate if the receptor is in the inactive conformation or the active conformation.
(PDF)

S6 Fig The correlation between the bilayer thickness and the lipid order for the high receptor density simulations of inactive (left) or activated (right) MOR with the BB beads of the receptors fixed. For each bin, the average order is plotted vs. the average thickness.
(PDF)

S7 Fig The bilayer thickness (nm, top row) and lipid order (bottom row) of the non-flipping lipids within 3.125 nm of the protein center of mass calculated as a function of the helix index during the last 2 μs of membrane equilibration of the low receptor density simulations with the BB beads of the receptors kept fixed. For the thickness, the average and standard error are indicated by a black line and a grey band, respectively. The overall average value of the order is indicated by red dashed lines in the bottom panels. The location of the center of mass of the helices is indicated by the vertical lines and TMs 1 through 7 are colored in blue, red, grey, orange, yellow, green, and pink, respectively. Because of the large tilt of TM3, its center of mass appears to the right of TM4.
(PDF)

S8 Fig The bilayer thickness (nm, top row) and lipid order (bottom row) of the non-flipping lipids within 3.125 nm of the protein center of mass calculated as a function of the helix index during the last 2 μs of membrane equilibration of the high receptor density simulations with the BB beads of the receptors kept fixed. For the thickness, the average and standard error are indicated by a black line and a grey band, respectively. The overall average value of the order is indicated by a red dashed lines in the bottom panels. The location of the center of mass of the helices is indicated by the vertical lines and TMs 1 through 7 are colored in blue, red, grey, orange, yellow, green, and pink, respectively. Because of the large tilt of TM3, its center of mass appears to the right of TM4.
(PDF)

S9 Fig Tilting of the principal axis of the active or inactive MOR in the low receptor density simulations with either restrained BB beads or freely moving receptors. The angle θ (radial coordinate, measuring the amount of tilting) is the angle between the protein principal axis and the normal to the membrane, while φ (polar angle, representing the direction of the tilting) is the angle between the projection of the principal axis on the xy plane and the projection of the vector connecting the center of mass of the protein with the center of mass of helix TM1.
(PDF)

S10 Fig Normalized distribution of lipid order during the final 2 μs of membrane equilibration for lipids near inactive MOR (blue) or active MOR (red) in the high receptor density simulations with the BB beads of the receptors kept fixed. The thick lines are the average of the five individual runs which are shown as thin lines. The individual runs are an average over all 16 protomers in the protein array.
(PDF)

S11 Fig Plots showing the average the lipid order as a function of time for one of the runs in the high receptor density simulations of inactive (top row) or active (bottom row) MORs. The data is averaged over all lipids, excluding the ones that flip-flop across the membrane (i.e. CHOL, CER, and DAG). A value of 0 (orange color) indicates a fully ordered lipid tail, and the larger the value, the more disordered the tail. In both cases, the white color of the color bar is set to the average value of the simulations with the inactive receptors. The centers of mass of the seven TM helices are indicated by the colored dots as follows: TMs 1 through 7 are colored in blue, red, grey, orange, yellow, green, and pink, respectively.
(PDF)

S12 Fig Average lipid thickness (upper triangle) and order (lower triangle) calculated as a function of the protein-protein distance *r* and the lipid position *d* for the following interfaces: TM5/TM5 and TM1,2,H8/TM1,2,H8 for the active MOR (panels A and B, respectively), and TM1,2,H8/TM5 for the inactive or active MOR (panels C and D, respectively). Trajectories from both the high and low receptor density simulations with the receptors free to move were used to generate the plots.
(PDF)

S13 Fig Probability distribution of CHOL order in the headgroup region of the membrane (dashed line) and the hydrophobic part of the membrane (solid lines) during the low receptor density simulations in which the BB beads of the receptors were kept fixed. (PDF)

S14 Fig Residence time of PC (left) or cholesterol (right) during the production runs of the membrane with high receptor density. The error bars represent the deviation over the five runs.
(PDF)

S15 Fig The z-coordinate of the CHOL’s ROH or CER/DAG’s AM1 or GL1 beads as a function of the minimum distance to the BB beads of the protein for inactive MORs (top row) or active MORs (bottom row) during the final 2 μs membrane equilibration in the simulations with high receptor density and the BB beads of the receptors kept fixed. (PDF)

S16 Fig The z-coordinate of the AM1 or GL1 beads as a function of lipid order for the DAG (top row) or CER (bottom row) lipids in the plasma membrane with inactive MOR, active MOR, or no receptors. The data in the plots for the membranes with receptors are from the final 2 μs membrane equilibration in the simulations with high receptor density and the BB beads of the receptors fixed.
(PDF)

S17 Fig Kinetic network of the cholesterol movement in the z-direction as a function of the distance from the protein for the A) inactive and B) active MORs generated with the plot_network routine of pyemma using the final 2 μs of membrane equilibration of the simulations with high receptor density and the BB beads of the receptors fixed. The initial geometric clustering was performed using the contacts formed between a single cholesterol molecule and the residues of the protein. The five pathways with the highest fluxes are shown with the thickness of the arrows indicative of the relative flux. The size of the circle is proportional to the size of the state. The states are colored according to their average order with orange corresponding to a cholesterol oriented parallel to the membrane normal and dark purple representing a perpendicular orientation.
(PDF)

S18 Fig Average of the number of isolated receptors calculated as a function of time during the production runs of the membrane with high receptor density. Values for the simulations with inactive or active receptors are reported in blue and red, respectively.
(PDF)

S19 Fig Model structures of the A) inactive/inactive MORs interacting at the TM1,2,H8/TM4 interface and B) active/active MORs interacting at the TM1,2,H8/TM1,2,H8 interface during the final μs of the high density simulations in which the BB beads of the receptors were kept fixed. Helices involved in the interface are colored by frequency of interaction with cholesterol (white to blue to green indicates low to high probability).
(PDF)

S20 Fig Snapshot of the production runs of the simulations with high receptor density at 0, 5, 10, and 20 μs for the A) inactive, B) active, and C) mixed arrays of MOR. The lipids are in grey, the inactive proteins in blue, and the active proteins are red.
(PDF)

S1 Movie Movie showing the average of the lipid order as a function of time for one run with inactive receptors in the membrane with high receptor density. The snapshots in S11 Fig were taken from this trajectory. The data is averaged over all lipids, excluding the ones that flip-flop across the membrane (i.e. CHOL, CER, and DAG). A value of 0 (orange color) indicates a fully ordered lipid tail, and the larger the value, the more disordered the tail. In both cases, the white color of the color bar is set to the average value of the simulations with the inactive receptors. The center of mass of the seven TM helices are indicated by the colored dots as follows: TMs 1 through 7 are colored in blue, red, grey, orange, yellow, green, and pink, respectively.
(MP4)

## References

[1] Gimpl G, Fahrenholz F. Cholesterol as stabilizer of the oxytocin receptor. Biochim Biophys Acta—Biomembr. 2002;1564: 384–392. 10.1016/s0005-2736(02)00475-3 12175921

[2] Hanson MA, Cherezov V, Griffith MT, Roth CB, Jaakola VP, Chien EYT, et al A specific cholesterol binding site is established by the 2.8 Å structure of the human β2-adrenergic receptor. Structure. 2008;16: 897–905. 10.1016/j.str.2008.05.001 18547522 PMC2601552

[3] Gimpl G, Burger K, Fahrenholz F. Cholesterol as modulator of receptor function. Biochemistry. 1997;36: 10959–10974. 10.1021/bi963138w 9283088

[4] Pucadyil TJ, Chattopadhyay A. Cholesterol modulates ligand binding and G-protein coupling to serotonin1A receptors from bovine hippocampus. Biochim Biophys Acta—Biomembr. 2004;1663: 188–200. 10.1016/j.bbamem.2004.03.010 15157621

[5] Zheng Pearsall, Hurst Zhang, Chu Zhou, et al Palmitoylation and membrane cholesterol stabilize μ-opioid receptor homodimerization and G protein coupling. BMC Cell Biol. 2012;13: 6 10.1186/1471-2121-13-6 22429589 PMC3317874

[6] Prasanna X, Chattopadhyay A, Sengupta D. Cholesterol modulates the dimer interface of the β2-adrenergic receptor via cholesterol occupancy sites. Biophys J. Elsevier; 2014;106: 1290–1300. 10.1016/j.bpj.2014.02.002 PMC3984991 24655504

[7] Oates J, Faust B, Attrill H, Harding P, Orwick M, Watts A. The role of cholesterol on the activity and stability of neurotensin receptor 1. Biochim Biophys Acta—Biomembr. 2012;1818: 2228–2233. 10.1016/j.bbamem.2012.04.010 22551944

[8] Paila YD, Kombrabail M, Krishnamoorthy G, Chattopadhyay A. Oligomerization of the serotonin 1A receptor in live cells: A time-resolved fluorescence anisotropy approach. J Phys Chem B. 2011;115: 11439–11447. 10.1021/jp201458h 21866959

[9] Gibson NJ, Brown MF. Lipid headgroup and acyl chain composition modulate the MI-MI1 equilibrium of rhodopsin in recombinant membranes. Biochemistry. 1993;32: 2438–2454. 8443184 10.1021/bi00060a040

[10] Dawaliby R, Trubbia C, Delporte C, Masureel M, Van Antwerpen P, Kobilka BK, et al Allosteric regulation of G protein–coupled receptor activity by phospholipids. Nat Chem Biol. 2016;12: 35–41. 10.1038/nchembio.1960 26571351 PMC4718399

[11] Inagaki S, Ghirlando R, White JF, Gvozdenovic-Jeremic J, Northup JK, Grisshammer R. Modulation of the interaction between neurotensin receptor NTS1 and Gq protein by lipid. J Mol Biol. 2012;417: 95–111. 10.1016/j.jmb.2012.01.023 22306739 PMC3294418

[12] Killian JA. Hydrophobic mismatch between proteins and lipids in membranes. Biochim Biophys Acta—Rev Biomembr. 1998;1376: 401–415. 10.1016/s0304-4157(98)00017-3 9805000

[13] Lingwood D, Simons K. Lipid rafts as a membrane-organizing principle. Science. 2010;327: 46–50. 10.1126/science.1174621 20044567

[14] Sonnino S, Prinetti A. Membrane domains and the “lipid raft” concept. Curr Med Chem. 2013;20: 4–21. 23150999

[15] Liu W, Chun E, Thompson AA, Chubukov P, Xu F, Katritch V, et al Structural basis for allosteric regulation of GPCRs by sodium ions. Science. 2012;337: 232–6. 10.1126/science.1219218 22798613 PMC3399762

[16] Jafurulla M, Chattopadhyay A. Membrane lipids in the function of serotonin and adrenergic receptors. Curr Med Chem. 2013;20: 47–55. 23151002

[17] Cherezov V, Rosenbaum DM, Hanson MA, Rasmussen SGF, Thian FS, Kobilka TS, et al High-resolution crystal structure of an engineered human β2-adrenergic G-protein couped receptor. Science. 2007;318: 1258–1266. 10.1126/science.1150577 17962520 PMC2583103

[18] Wu H, Wang C, Gregory KJ, Han GW, Cho HP, Xia Y, et al Structure of a class C GPCR metabotropic glutamate receptor 1 bound to an allosteric modulator. Science. 2014;344: 58–64. 10.1126/science.1249489 24603153 PMC3991565

[19] Periole X, Huber T, Marrink S, Sakmar TP. G protein-coupled receptors self-assemble in dynamics simulations of model bilayers. J Am Chem Soc. 2007;129: 10126–10132. 10.1021/ja0706246 17658882

[20] Soubias O, Teague WE, Hines KG, Mitchell DC, Gawrisch K. Contribution of membrane elastic energy to rhodopsin function. Biophys J. Biophysical Society; 2010;99: 817–824. 10.1016/j.bpj.2010.04.068 PMC2913204 20682259

[21] Pitman MC, Grossfield A, Suits F, Feller SE. Role of cholesterol and polyunsaturated chains in lipid − protein interactions : Molecular dynamics simulation of rhodopsin in a realistic membrane environment. Biochim Biophys Acta. 2005; 4576–4577. 10.1021/ja042715y 15796514

[22] Grossfield A, Feller SE, Pitman MC. A role for direct interactions in the modulation of rhodopsin by omega-3 polyunsaturated lipids. Proc Natl Acad Sci U S A. 2006;103: 4888–4893. 10.1073/pnas.0508352103 16547139 PMC1458765

[23] Lee JY, Patel R, Lyman E. Ligand-dependent cholesterol interactions with the human A2A adenosine receptor. Chem Phys Lipids. Elsevier Ireland Ltd; 2013;169: 39–45. 10.1016/j.chemphyslip.2013.02.002 23454349 PMC3652319

[24] Khelashvili G, Grossfield A, Feller SE, Pitman MC, Weinstein H. Structural and dynamic effects of cholesterol at preferred sites of interaction with rhodopsin identified from microsecond length molecular dynamics simulations. Proteins Struct Funct Bioinforma. 2009;76: 403–417. 10.1002/prot.22355 PMC4101808 19173312

[25] Sengupta D, Chattopadhyay A. Identification of Cholesterol Binding Sites in the Serotonin1A Receptor. J Phys Chem B. 2012;116: 12991–12996. 10.1021/jp309888u 23067252

[26] Lyman E, Higgs C, Kim B, Lupyan D, Shelley JC, Farid R, et al A role for a specific cholesterol interaction in stabilizing the apo configuration of the human A2A adenosine receptor. Structure. Elsevier Ltd; 2009;17: 1660–1668. 10.1016/j.str.2009.10.010 PMC2796422 20004169

[27] Ingólfsson HI, Melo MN, Eerden FJ Van, Arnarez C, López CA, Wassenaar TA, et al Lipid organization of the plasma membrane. J Am Chem Soc. 2014;136: 14554–14559. 10.1021/ja507832e 25229711

[28] Provasi D, Boz MB, Johnston JM, Filizola M. Preferred supramolecular organization and dimer interfaces of opioid receptors from simulated self-association. PLoS Comput Biol. 2015;11: 1–21. 10.1371/journal.pcbi.1004148 PMC4379167 25822938

[29] Manglik A, Kruse AC, Kobilka TS, Thian FS, Mathiesen JM, Sunahara RK, et al Crystal structure of the μ-opioid receptor bound to a morphinan antagonist. Nature. 2012;485: 321–326. 10.1038/nature10954 22437502 PMC3523197

[30] Huang W, Manglik A, Venkatakrishnan AJ, Laeremans T, Feinberg EN, Sanborn AL, et al Structural insights into μ-opioid receptor activation. Nature. 2015;524: 315–21. 10.1038/nature14886 26245379 PMC4639397

[31] Gomes I, Fujita W, Gupta A, Saldanha SA, Negri A, Pinello CE, et al Identification of a μ-δ opioid receptor heteromer-biased agonist with antinociceptice activity. Proc Natl Acad Sci U S A. 2013;110: 12072–12077. 10.1073/pnas.1222044110 23818586 PMC3718106

[32] Zheng H, Chu J, Qiu Y, Loh HH, Law P-Y. Agonist-selective signaling is determined by the receptor location within the membrane domains. Proc Natl Acad Sci U S A. 2008;105: 9421–9426. 10.1073/pnas.0802253105 18599439 PMC2453714

[33] Moulédous L, Merker S, Neasta J, Roux B, Zajac JM, Mollereau C. Neuropeptide FF-sensitive confinement of mu opioid receptor does not involve lipid rafts in SH-SY5Y cells. Biochem Biophys Res Commun. 2008;373: 80–84. 10.1016/j.bbrc.2008.05.174 18544342

[34] Halls ML, Yeatman HR, Nowell CJ, Thompson G, Gondin AB, Civciristov S, et al Plasma membrane localization of the mu-opioid receptor controls spatiotemporal signaling. Sci Signal. 2016;9: ra16 10.1126/scisignal.aac9177 26861044

[35] Gaibelet G, Millot C, Lebrun C, Ravault S, Sauliere A, Andre A, et al Cholesterol content drives distinct pharmacological behaviours of μ-opioid receptor in different microdomains of the CHO plasma membrane. Mol Membr Biol. 2008;25: 423–435. 10.1080/09687680802203380 18651319

[36] Qiu Y, Wang Y, Law P, Chen H, Loh HH. Cholesterol regulates μ-opioid receptor-induced β-Arrestin 2 translocation to membrane lipid rafts. Mol Pharmacol. 2011;80: 210–218. 10.1124/mol.110.070870 21518774 PMC3127540

[37] Gu R, Ingólfsson HI, Vries AH De, Marrink SJ, Tieleman DP. Ganglioside-lipid and ganglioside-protein interactions revealed by coarse-grained and atomistic molecular dynamics simulations. J Phys Chem B. 2016; in press. 10.1021/acs.jpcb.6b07142 PMC5402298 27610460

[38] Hamilton JA. Fast flip-flop of cholesterol and fatty acids in membranes: implications for membrane transport proteins. Curr Opin Lipidol. 2003;14: 263–271. 10.1097/01.mol.0000073507.41685.9b 12840657

[39] Niemelä PS, Miettinen MS, Monticelli L, Hammaren H, Bjelkmar P, Murtola T, et al Membrane proteins diffuse as dynamic complexes with lipids. J Am Chem Soc. 2010;132: 7574–7575. 10.1021/ja101481b 20469857

[40] Koldsø H, Sansom MSP. Organization and dynamics of receptor proteins in a plasma membrane. J Am Chem Soc. 2015;137: 14694–14704. 10.1021/jacs.5b08048 26517394 PMC5591644

[41] Guixà-González R, Javanainen M, Gómez-Soler M, Cordobilla B, Domingo JC, Sanz F, et al Membrane omega-3 fatty acids modulate the oligomerisation kinetics of adenosine A2A and dopamine D2 receptors. Sci Rep. 2016;6: 19839 10.1038/srep19839 26796668 PMC4726318

[42] Periole Knepp, Sakmar Marrink, Huber. Structural determinants of the supramolecular organization of g protein-coupled receptors in bilayers. J Am Chem Soc. 2012;134: 10959–10965. 10.1021/ja303286e 22679925 PMC3406292

[43] Holdbrook DA, Huber RG, Piggot TJ, Bond PJ, Khalid S. Dynamics of crowded vesicles: Local and global responses to membrane composition. PLoS One. 2016;11: e0156963 10.1371/journal.pone.0156963 27310814 PMC4910979

[44] Mondal S, Johnston JM, Wang H, Khelashvili G, Filizola M, Weinstein H. Membrane driven spatial organization of GPCRs. Sci Rep. 2013;3: 2909 10.1038/srep02909 24105260 PMC3793225

[45] Sengupta D, Chattopadhyay A. Molecular dynamics simulations of GPCR–cholesterol interaction : An emerging paradigm. Biochim Biophys Acta—Biomembr. 2015;1848: 1775–1782. 10.1016/j.bbamem.2015.03.018 25817549

[46] Katira S, Mandadapu KK, Vaikuntanathan S, Smit B, Chandler D. Pre-transition effects mediate forces of assembly between transmembrane proteins. Elife. 2016;5: e13150 10.7554/eLife.13150 26910009 PMC4841784

[47] van Meer G, Voelker DR, Feigenson GW. Membrane lipids: where they are and how they behave. Nat Rev Mol Cell Biol. 2008;9: 112–124. 10.1038/nrm2330 18216768 PMC2642958

[48] Simons K, Toomre D. Lipid rafts and signal transduction. Nat Rev Mol Cell Biol. 2000;1: 31–39. 10.1038/35036052 11413487

[49] Ostrom RS, Insel PA. The evolving role of lipid rafts and caveolae in G protein-coupled receptor signaling: implications for molecular pharmacology. Br J Pharmacol. 2004;143: 235–45. 10.1038/sj.bjp.0705930 15289291 PMC1575337

[50] Goren MA, Morizumi T, Menon I, Joseph JS, Dittman JS, Cherezov V, et al Constitutive phospholipid scramblase activity of a G protein-coupled receptor. Nat Commun. 2014;5: 1–10. 10.1038/ncomms6115 PMC4198942 25296113

[51] Bennett WFD, MacCallum JL, Tieleman DP. Thermodynamic analysis of the effect of cholesterol on dipalmitoylphosphatidylcholine lipid membranes. J Am Chem Soc. 2009;131: 1972–1978. 10.1021/ja808541r 19146400

[52] Harroun TA, Katsaras J, Wassall SR. Cholesterol is found to reside in the center of a polyunsaturated lipid membrane. Biochemistry. 2008;47: 7090–7096. 10.1021/bi800123b 18543943

[53] Marrink SJ, De Vries AH, Harroun TA, Katsaras J, Wassall SR. Cholesterol shows preference for the interior of polyunsaturated lipid membranes. J Am Chem Soc. 2008;130: 10–11. 10.1021/ja076641c 18076174

[54] Jo S, Rui H, Lim JB, Klauda JB, Im W. Cholesterol flip-flop: Insights from free energy simulation studies. J Phys Chem B. 2010;114: 13342–13348. 10.1021/jp108166k 20923227

[55] Choubey A, Kalia RK, Malmstadt N, Nakano A, Vashishta P. Cholesterol translocation in a phospholipid membrane. Biophys J. 2013;104: 2429–2436. 10.1016/j.bpj.2013.04.036 23746515 PMC3672867

[56] Neale C, Herce HD, Pomès R, García AE. Can specific protein-lipid interactions stabilize an active state of the beta 2 adrenergic receptor? Biophys J. 2015;109: 1652–1662. 10.1016/j.bpj.2015.08.028 26488656 PMC4624154

[57] Logothetis DE, Petrou VI, Zhang M, Mahajan R, Meng X-Y, Adney SK, et al Phosphoinositide control of membrane protein function: A frontier led by studies on ion channels. Annu Rev Physiol. 2015;77: 81–104. 10.1146/annurev-physiol-021113-170358 25293526 PMC4485992

[58] Mancia F, Assur Z, Herman AG, Siegel R, Hendrickson WA. Ligand sensitivity in dimeric associations of the serotonin 5HT2c receptor. EMBO Rep. 2008;9: 363–9. 10.1038/embor.2008.27 18344975 PMC2271072

[59] Guo W, Shi L, Filizola M, Weinstein H, Javitch JA. Crosstalk in G protein-coupled receptors: changes at the transmembrane homodimer interface determine activation. Proc Natl Acad Sci U S A. 2005;102: 17495–17500. 10.1073/pnas.0508950102 16301531 PMC1287488

[60] Xue L, Rovira X, Scholler P, Zhao H, Liu J, Pin J-P, et al Major ligand-induced rearrangement of the heptahelical domain interface in a GPCR dimer. Nat Chem Biol. ing Group; 2015;11: 134–140. 10.1038/nchembio.1711 25503927

[61] Fenalti G, Giguere PM, Katritch V, Huang X-P, Thompson AA, Cherezov V, et al Molecular control of δ-opioid receptor signalling. Nature. 2014;506: 191–196. 10.1038/nature12944 24413399 PMC3931418

[62] Huang J, Chen S, Zhang JJ, Huang X-Y. Crystal structure of oligomeric β1-adrenergic G protein-coupled receptors in ligand-free basal state. Nat Struct Mol Biol. 2013;20: 419–25. 10.1038/nsmb.2504 23435379 PMC3618578

[63] Wu B, Chien EYT, Mol CD, Fenalti G, Liu W, Katritch V, et al Structures of the CXCR4 chemokine GPCR with small-molecule and cyclic peptide antagonists. Science. 2010;330: 1066–1071. 10.1126/science.1194396 20929726 PMC3074590

[64] Tan Q, Zhu Y, Li J, Chen Z, Han GW, Kufareva I, et al Structure of the CCR5 chemokine receptor-HIV entry inhibitor maraviroc complex. Science. 2013;341: 1387–1390. 10.1126/science.1241475 24030490 PMC3819204

[65] Parsadaniantz SM, Rostene W, Goazigo ARL. Opioid and chemokine receptor crosstalk: a promising target for pain therapy? Nat Rev Neurosci. 2015;16: 69–78. 10.1038/nrn3858 25588373

[66] Sali A, Blundell TL. Comparative protein modelling by satisfaction of spatial restraints. J Mol Biol. 1993;234: 779–815. 10.1006/jmbi.1993.1626 8254673

[67] Marrink S, Vries A De, Mark A. Coarse grained model for semiquantitative lipid simulations. J Phys Chem B. 2004;108: 750–760.

[68] Monticelli L, Kandasamy SK, Periole X, Larson RG, Tieleman DP, Marrink SJ. The MARTINI coarse grained force field: extension to proteins. J Chem Theory Comput. 2008;4: 819–834. 10.1021/ct700324x 26621095

[69] Marrink SJ, Risselada HJ, Yefimov S, Tieleman DP, Vries AH De. The MARTINI force field : Coarse grained model for biomolecular simulations. J Phys Chem B. 2007;111: 7812–7824. 10.1021/jp071097f 17569554

[70] Periole X, Cavalli M, Marrink SJ, Ceruso MA. Combining an elastic network with a coarse-grained molecular force field: Structure, dynamics, and intermolecular recognition. J Chem Theory Comput. 2009;5: 2531–2543. 10.1021/ct9002114 26616630

[71] Provasi D, Johnston JM, Filizola M. Lessons from free energy simulations of δ-opioid receptor homodimers involving the fourth transmembrane helix. Biochemistry. 2010;49: 6771–6776. 10.1021/bi100686t 20617813 PMC2914489

[72] Ballesteros J, Weinstein H. Integrated methods for the construction of three-dimensional models and computational probing of structure-function relations in G protein coupled receptors. Methods Neurosci. 1995;25: 366–428.

[73] Wassenaar TA, Ingólfsson HI, Böckmann RA, Tieleman DP, Marrink SJ. Computational lipidomics with insane: A versatile tool for generating custom membranes for molecular simulations. J Chem Theory Comput. 2015;11: 2144–2155. 10.1021/acs.jctc.5b00209 26574417

[74] Bussi G, Donadio D, Parrinello M. Canonical sampling through velocity rescaling. J Chem Phys. 2007;126: 14101. 10.1063/1.2408420 17212484

[75] Berensen HJC, Postma JPM, van Gunsteren WF, DiNola A, Haak JR. Molecular dynamics with coupling to an external bath. J Chem Phys. 1984;81: 3684–3690.

[76] Pronk S, Páll S, Schulz R, Larsson P, Bjelkmar P, Apostolov R, et al GROMACS 4.5: A high-throughput and highly parallel open source molecular simulation toolkit. Bioinformatics. 2013;29: 845–54. 10.1093/bioinformatics/btt055 23407358 PMC3605599

[77] Hess B, Kutzner C, Spoel D Van Der, Lindahl E. GROMACS 4: Algorithms for highly efficient, load-balanced, and scalable molecular simulation. J Chem Theory Comput. 2008;4: 435–447. 10.1021/ct700301q 26620784

[78] Scherer MK, Trendelkamp-Schroer B, Paul F, Pérez-Hernández G, Hoffmann M, Plattner N, et al PyEMMA 2: A software package for estimation, validation, and analysis of markov models. J Chem Theory Comput. 2015;11: 5525–5542. 10.1021/acs.jctc.5b00743 26574340

[79] Gelman A, Lee D, Guo J. Stan : A Probabilistic Programming Language for Bayesian Inference and Optimization. J Educ Behav Stat. 2015;40: 530–543.

[80] Michaud-Agrawal N, Denning EJ, Woolf TB, Beckstein O. MDAnalysis: A toolkit for the analysis of molecular dynamics simulations. J Comput Chem. 2011;32: 2319–2327. 10.1002/jcc.21787 21500218 PMC3144279

