# Supplementary material for: Impact of Lipid Composition and Receptor Conformation on the Spatio-temporal Organization of μ-Opioid Receptors in a Multi-component Plasma Membrane Model
Source: PLoS Comput Biol. 2016 Dec 13;12(12):e1005240. doi: 10.1371/journal.pcbi.1005240 (PMC5154498; doi:10.1371/journal.pcbi.1005240)
Supplement: S1 Table — All residues listed are in contact with either the inactive or active receptor at least 10% of the simulation time. Residues exhibiting the greatest difference between the two receptor conformations are shown in bold. (PDF) [file pcbi.1005240.s001.pdf]

Residue	Inactive	Active
V94 (1.58)	0.17	0.12
R95 (1.59)	0.15	0.10
R182 (4.40)	0.14	0.10
K185 (4.43)	0.13	0.11
**I256 (5.62)**	**0.15**	**0.01**
L257 (5.63)	0.14	0.22
**L259 (5.65)**	**0.10**	**0.00**
K260 (5.66)	0.20	0.25
**S261 (5.67)**	**0.06**	**0.28**
**R276 (6.31)**	**0.04**	**0.16**
R280 (6.35)	0.24	0.23
F338 (7.55)	0.16	0.13
L339 (7.56)	0.22	0.18
**K344 (H8)**	**0.02**	**0.10**
**F347 (H8)**	**0.02**	**0.11**
**R348 (H8)**	**0.03**	**0.11**
**C351 (H8)**	**0.03**	**0.10**
