# Supplementary material for: Impact of Lipid Composition and Receptor Conformation on the Spatio-temporal Organization of μ-Opioid Receptors in a Multi-component Plasma Membrane Model
Source: PLoS Comput Biol. 2016 Dec 13;12(12):e1005240. doi: 10.1371/journal.pcbi.1005240 (PMC5154498; doi:10.1371/journal.pcbi.1005240)
Supplement: S2 Table — The mean fraction of dimers belonging to each specific interface is reported along with its 97.5% credible intervals. The most frequently observed interfaces for each dimer type are highlighted in bold. (PDF) [file pcbi.1005240.s002.pdf]

Interface	Inactive MOR/ Inactive MOR	Active MOR/ Active MOR	Inactive MOR/ Active MOR
TM1,2,H8/TM1,2,H8	0.08 (0.07, 0.09)	**0.26 (0.24, 0.27)**	-
TM1,2,H8/TM4	**0.31 (0.30, 0.33)**	0.09 (0.08, 0.09)	-
TM1,2,H8/TM4,5	**0.15 (0.14, 0.16)**	-	-
TM1,2,H8/TM5	0.06 (0.05, 0.06)	**0.23 (0.22, 0.25)**	**0.61 (0.58,0.65)**
TM1,2,H8/TM5,6	-	**0.26 (0.24, 0.27)**	-
TM1,8/TM4	0.08 (0.07, 0.09)	-	-
TM1,H8/TM6,7	0.08 (0.07, 0.09)	-	-
TM2,H8/TM4	-	-	0.39 (0.35,0.42)
TM4/TM4	-	0.17 (0.16, 0.18)	-
TM4/TM5	0.08 (0.07, 0.09)	-	-
TM4/TM7	0.07 (0.07, 0.08)	-	-
TM5/TM5	0.08 (0.07, 0.09)	-	-
