# Supplementary material for: Impact of Lipid Composition and Receptor Conformation on the Spatio-temporal Organization of μ-Opioid Receptors in a Multi-component Plasma Membrane Model
Source: PLoS Comput Biol. 2016 Dec 13;12(12):e1005240. doi: 10.1371/journal.pcbi.1005240 (PMC5154498; doi:10.1371/journal.pcbi.1005240)
Supplement: S3 Table — Residues in contact with CHOL for more than 75% of the simulation time are in bold. (PDF) [file pcbi.1005240.s003.pdf]

**TM1,2,H8/ TM4**	**TM1,2,H8/ TM4,5**	**TM1,2,H8/ TM1,2,H8**	**TM1,2,H8/ TM5**	**TM1,2,H8/ TM5,6**
L116 (2.52)	A73 (1.37)	A73 (1.37)	M72 (1.36)	**A73 (1.37)**
**S119 (2.55)**	L74 (1.38)	L74 (1.38)	A73 (1.37)	**S76 (1.40)**
**T120 (2.56)**	S76 (1.40)	S76 (1.40)	L74 (1.38)	**I77 (1.41)**
V187 (4.45)	I77 (1.41)	**I77 (1.41)**	S76 (1.40)	C79 (1.43)
V202 (4.60)	L116 (2.52)	C79 (1.43)	I77 (1.41)	V80 (1.44)
	S119 (2.55)	V80 (1.44)	L116 (2.52)	V81 (1.45)
	T120 (2.56)	V81 (1.45)	**S119 (2.55)**	L116 (2.52)
	S196 (4.54)	L83 (1.47)	**T120 (2.56)**	**S119 (2.55)**
	A197 (4.55)	N109 (2.45)	F123 (2.59)	**P122 (2.58)**
	I238 (5.44)	L112 (2.48)	C235 (5.41)	I238 (5.44)
	F239 (5.45)	A113 (2.49)	**I238 (5.44)**	F241 (5.47)
	F241 (5.47)	**L116 (2.52)**	**F239 (5.45)**	**I242 (5.48)**
	**I242 (5.48)**	**S119 (2.55)**	F241 (5.47)	M243 (5.49)
	M243 (5.49)	T120 (2.56)	**I242 (5.48)**	V245 (5.51)
	V245 (5.51)	**P122 (2.58)**	M243 (5.49)	R280 (6.35)
	L246 (5.52)		V245 (5.51)	L283 (6.38)
	I256 (5.62)		L257 (5.63)	V284 (6.39)
	L257 (5.63)			I290 (6.45)
				**T294 (6.49)**
