# Supplementary figures and images for: Impact of Lipid Composition and Receptor Conformation on the Spatio-temporal Organization of μ-Opioid Receptors in a Multi-component Plasma Membrane Model
Source: PLoS Comput Biol. 2016 Dec 13;12(12):e1005240. doi: 10.1371/journal.pcbi.1005240 (PMC5154498; doi:10.1371/journal.pcbi.1005240)

A) CHOL

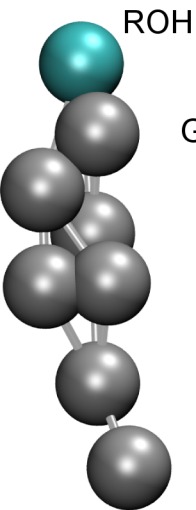

B) PC/SM

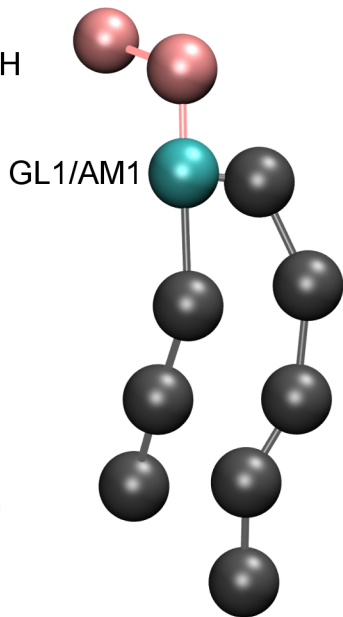

C)

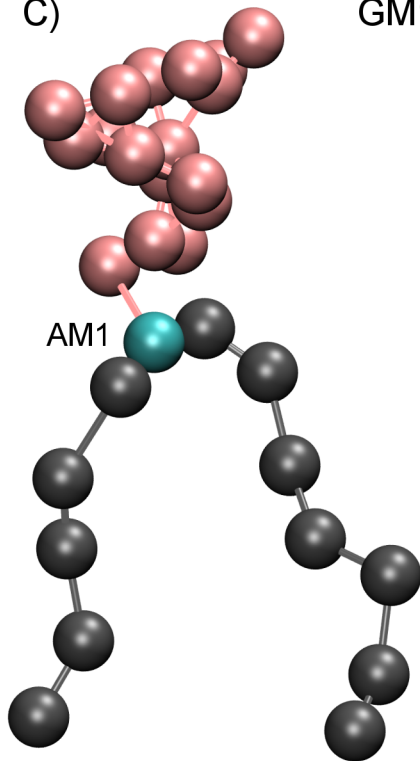

GM1

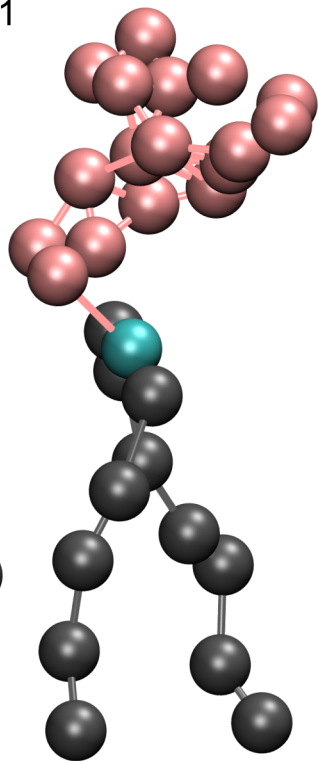

Supplement: S1 Fig — Structure of A) a cholesterol, B) a PC/SM lipid, and C) a GM lipid with the ROH, GL1, and AM1 beads used in the lipid analysis colored in cyan. The headgroup beads are in pink and the remaining tail beads in grey. (PDF) [file pcbi.1005240.s004.pdf]

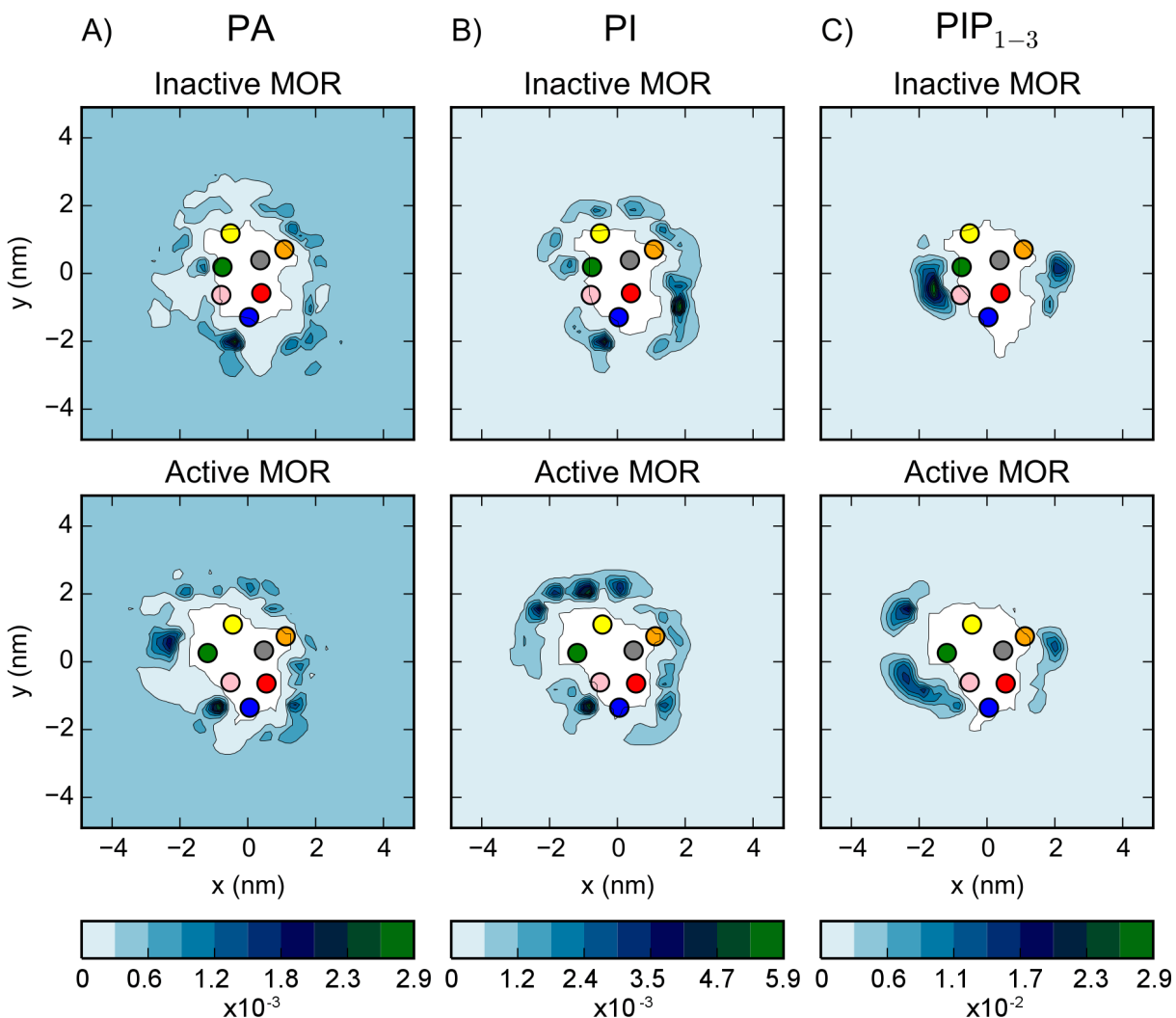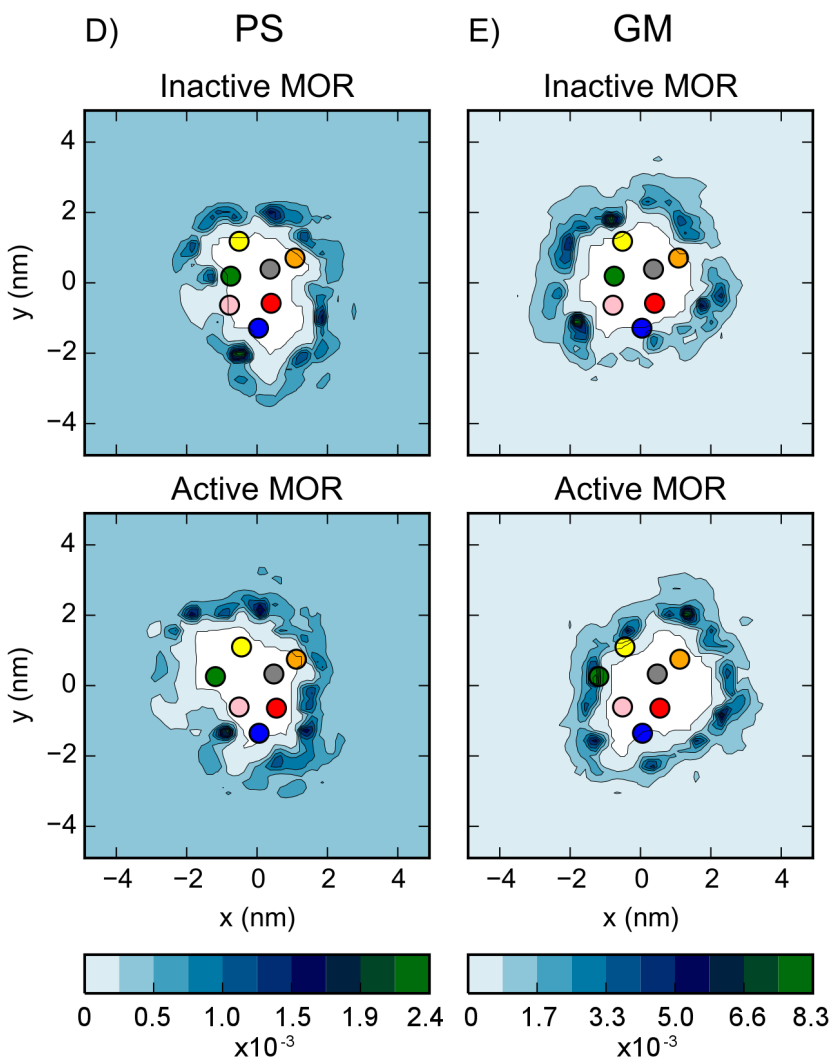

Supplement: S2 Fig — Lipid distributions of A) PA, B) PI, C) PIP1-3, D) PS, and E) GM around individual protomers during the final 2 μs of the low receptor density simulations with the BB beads of the receptors fixed. The dots indicate the centers of mass of the BB beads of the receptor helices. (PDF) [file pcbi.1005240.s005.pdf]

Upper Leaflet

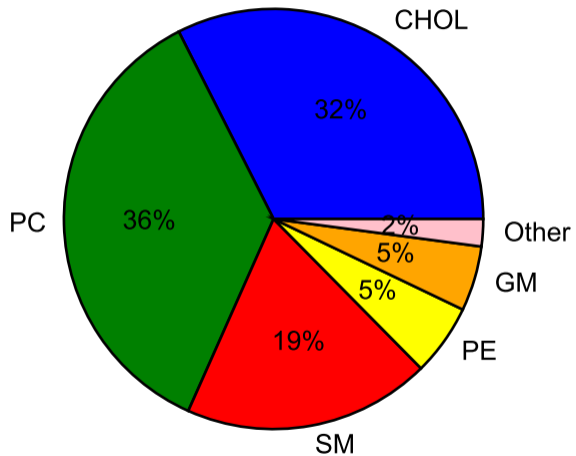

Lower Leaflet

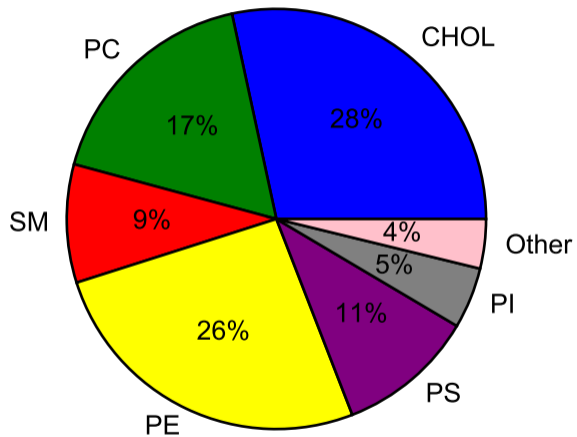

Supplement: S3 Fig — ‘Other’ in the upper leaflet includes: CER (0.7%), LPC (1.0%), and DAG (0.4%). ‘Other’ in the lower leaflet includes: PA (1.5%), PIP1-3 (1.6%), CER (0.1%), and DAG (0.5%). The total number of lipids in the upper and lower leaflets was approximately 805 and 750, respectively for the high receptor density membrane and 3200 and 3000 for the low receptor density membrane. (PDF) [file pcbi.1005240.s006.pdf]

Lipid Order Inactive/Active MOR

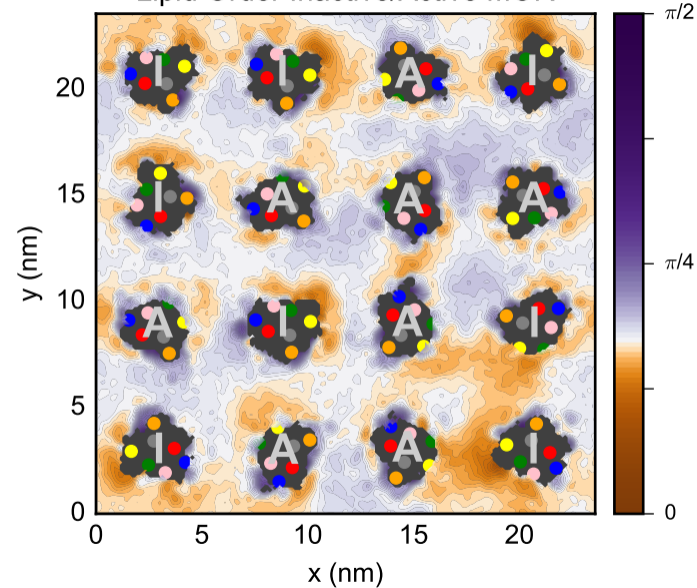

Bilayer Thickness Inactive/Active MOR

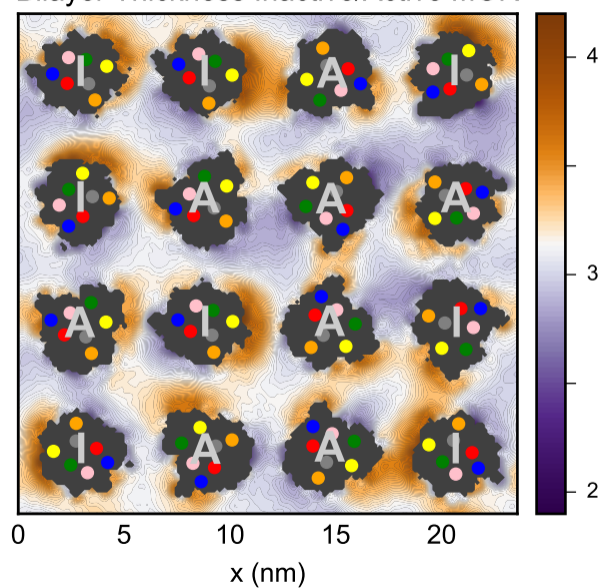

Supplement: S5 Fig — The data is averaged over all lipids, excluding the ones that flip-flop across the membrane (i.e. CHOL, CER, and DAG). For the order, a value of 0 indicates a fully ordered lipid tail, and the larger the value, the more disordered the tail. The units of thickness are nm. In both cases, the white color in the color bar is set to the average value of the simulations with the inactive receptors. The centers of mass of the seven TM helices are indicated by the colored dots as follows: TMs 1 through 7 are colored in blue, red, grey, orange, yellow, green, and pink, respectively. The ‘I’ and ‘A’ indicate if the receptor is in the inactive conformation or the active conformation. (PDF) [file pcbi.1005240.s008.pdf]

Inactive MOR

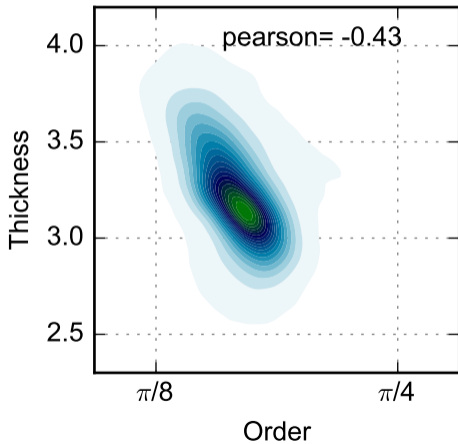

Active MOR

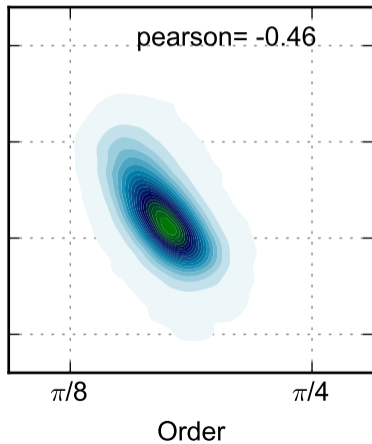

Supplement: S6 Fig — For each bin, the average order is plotted vs. the average thickness. (PDF) [file pcbi.1005240.s009.pdf]

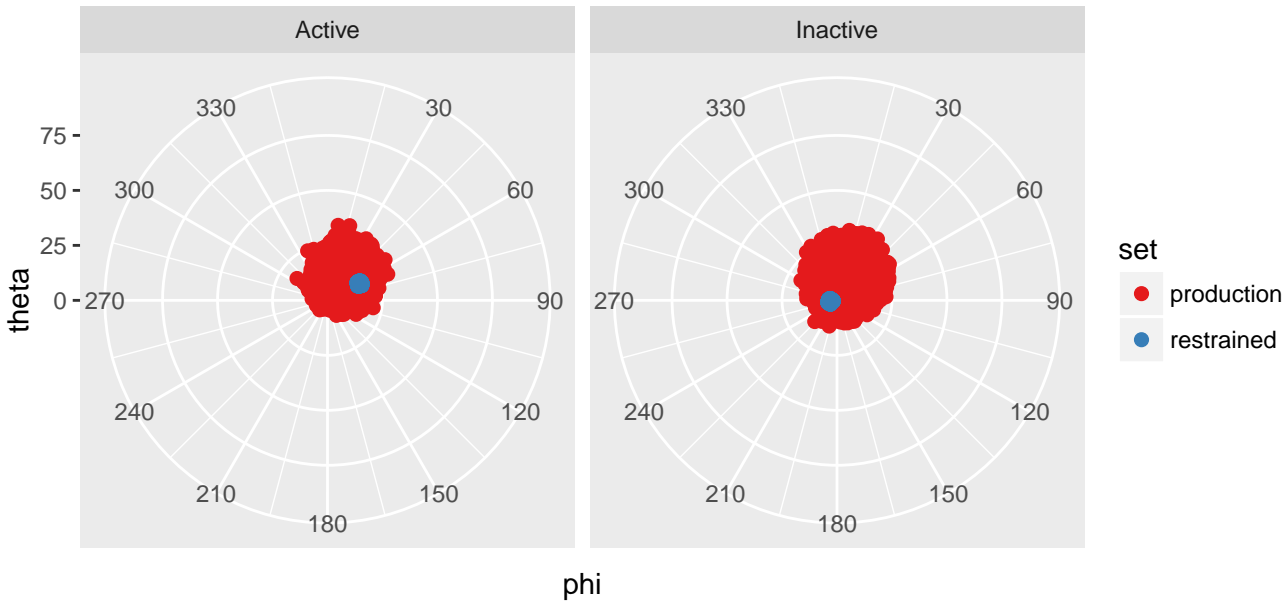

Supplement: S9 Fig — The angle θ (radial coordinate, measuring the amount of tilting) is the angle between the protein principal axis and the normal to the membrane, while φ (polar angle, representing the direction of the tilting) is the angle between the projection of the principal axis on the xy plane and the projection of the vector connecting the center of mass of the protein with the center of mass of helix TM1. (PDF) [file pcbi.1005240.s012.pdf]

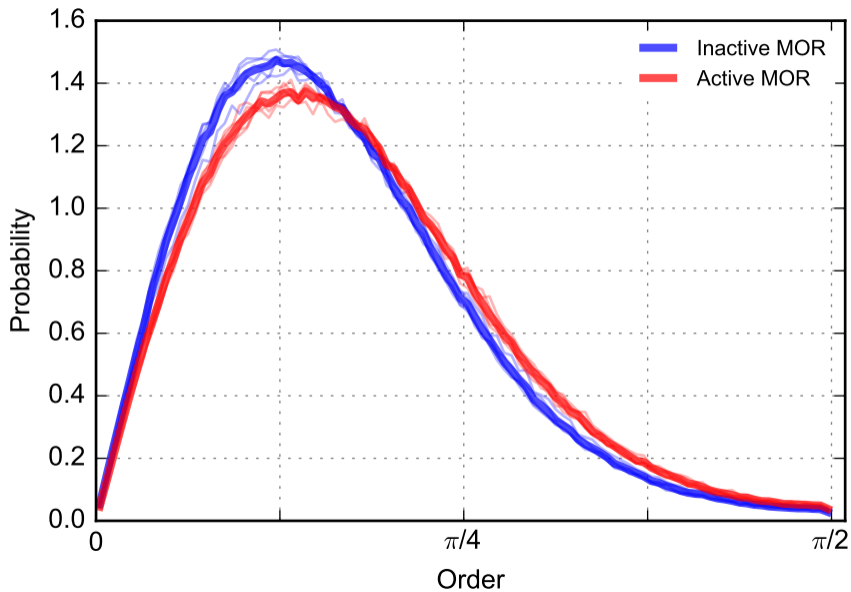

Supplement: S10 Fig — The thick lines are the average of the five individual runs which are shown as thin lines. The individual runs are an average over all 16 protomers in the protein array. (PDF) [file pcbi.1005240.s013.pdf]

INACTIVE MOR

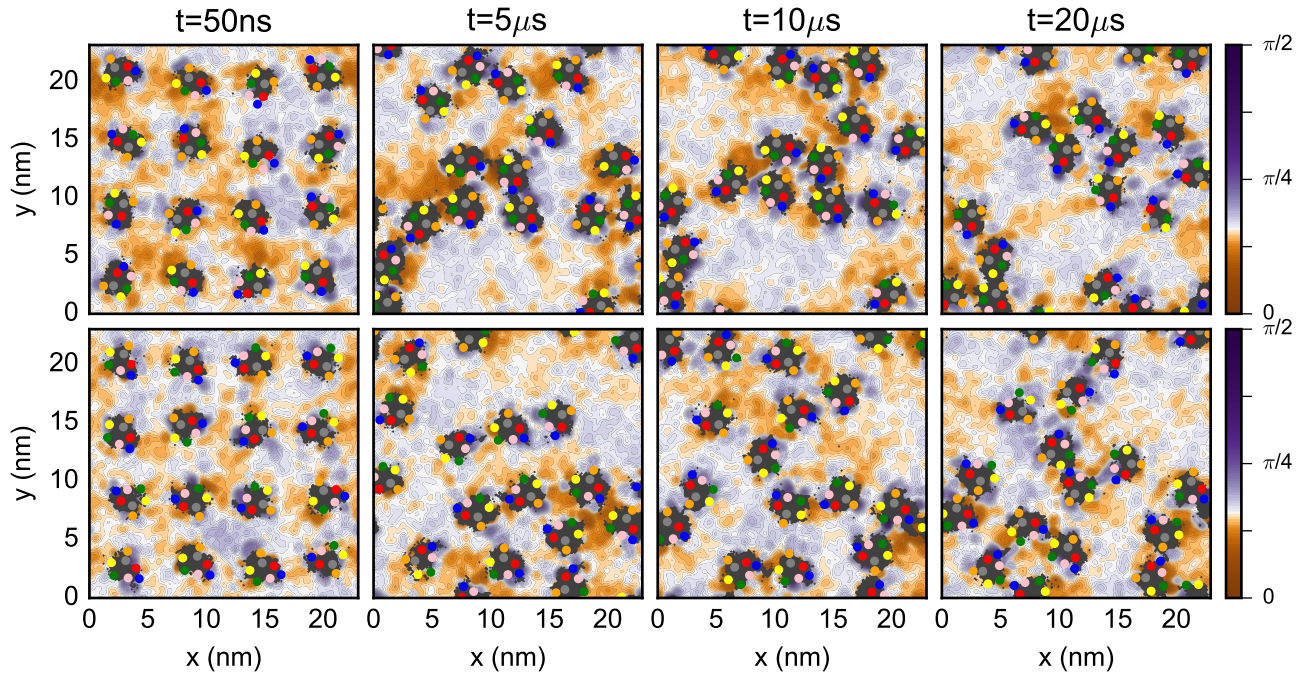

Supplement: S11 Fig — The data is averaged over all lipids, excluding the ones that flip-flop across the membrane (i.e. CHOL, CER, and DAG). A value of 0 (orange color) indicates a fully ordered lipid tail, and the larger the value, the more disordered the tail. In both cases, the white color of the color bar is set to the average value of the simulations with the inactive receptors. The centers of mass of the seven TM helices are indicated by the colored dots as follows: TMs 1 through 7 are colored in blue, red, grey, orange, yellow, green, and pink, respectively. (PDF) [file pcbi.1005240.s014.pdf]

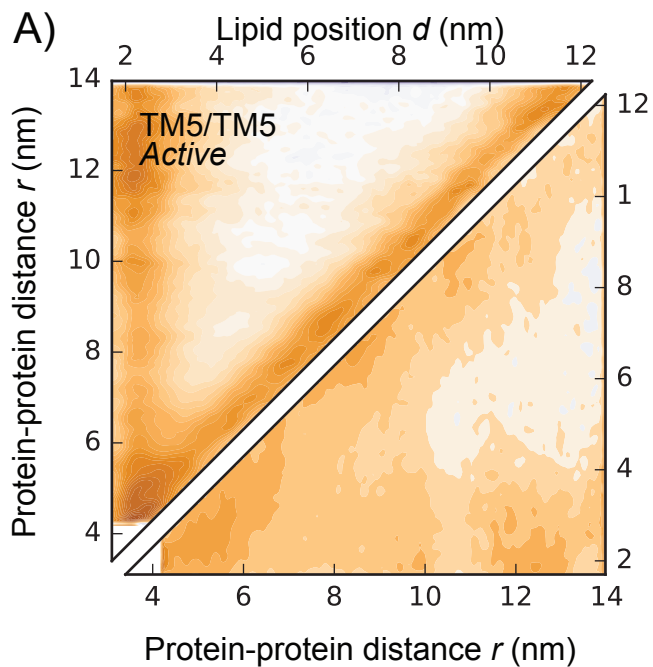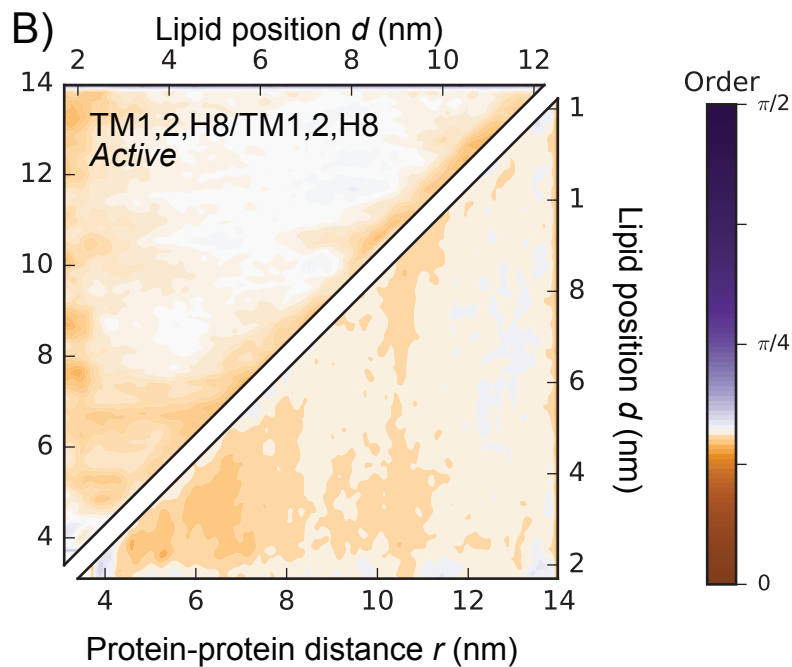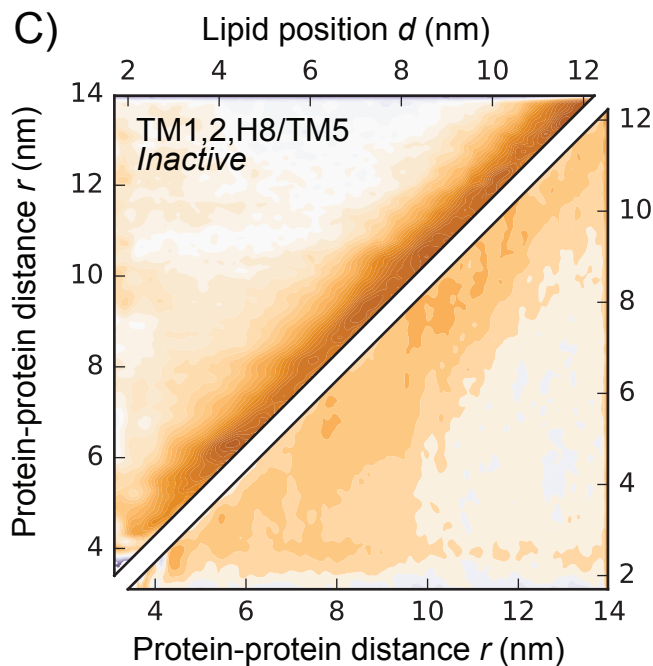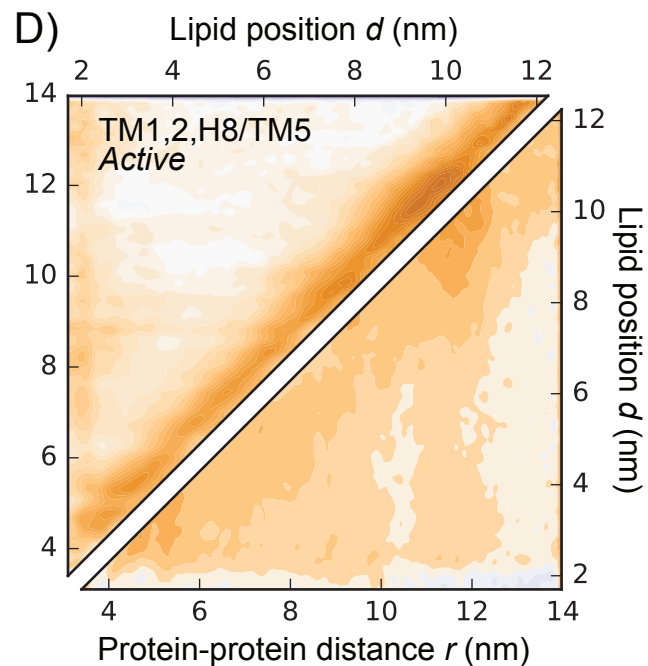

Supplement: S12 Fig — Average lipid thickness (upper triangle) and order (lower triangle) calculated as a function of the protein-protein distance r and the lipid position d for the following interfaces: TM5/TM5 and TM1,2,H8/TM1,2,H8 for the active MOR (panels A and B, respectively), and TM1,2,H8/TM5 for the inactive or active MOR (panels C and D, respectively). Trajectories from both the high and low receptor density simulations with the receptors free to move were used to generate the plots. (PDF) [file pcbi.1005240.s015.pdf]

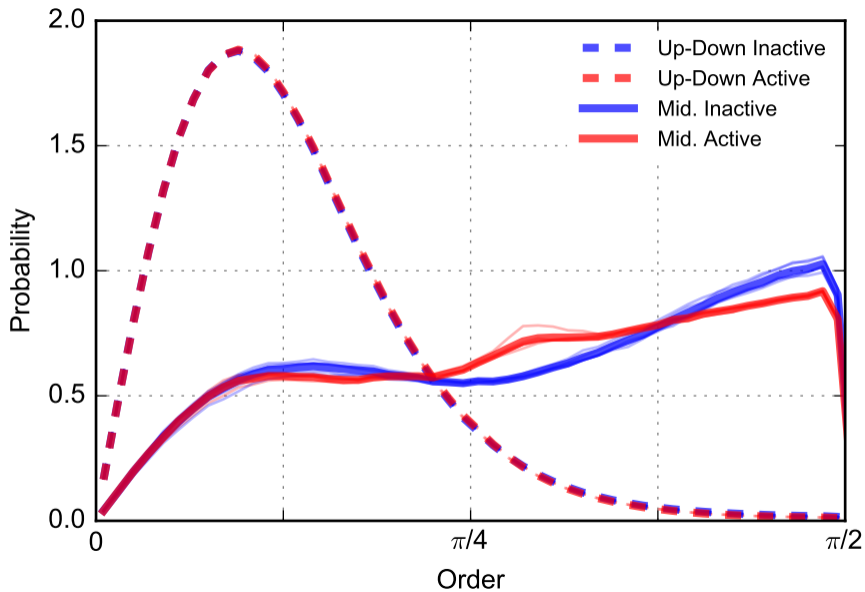

Supplement: S13 Fig — (PDF) [file pcbi.1005240.s016.pdf]

PC Mean Residence Times

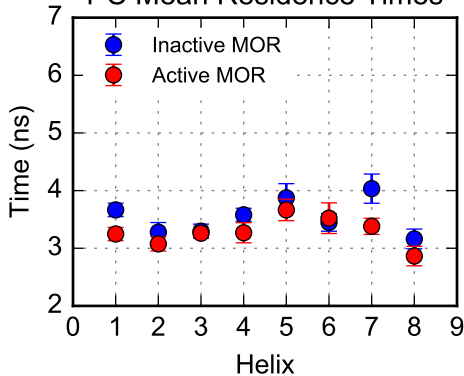

CHOL Mean Residence Times

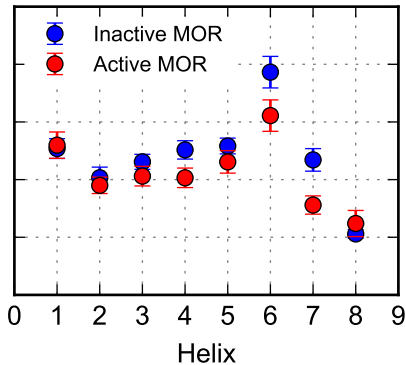

Supplement: S14 Fig — The error bars represent the deviation over the five runs. (PDF) [file pcbi.1005240.s017.pdf]

## INACTIVE MOR

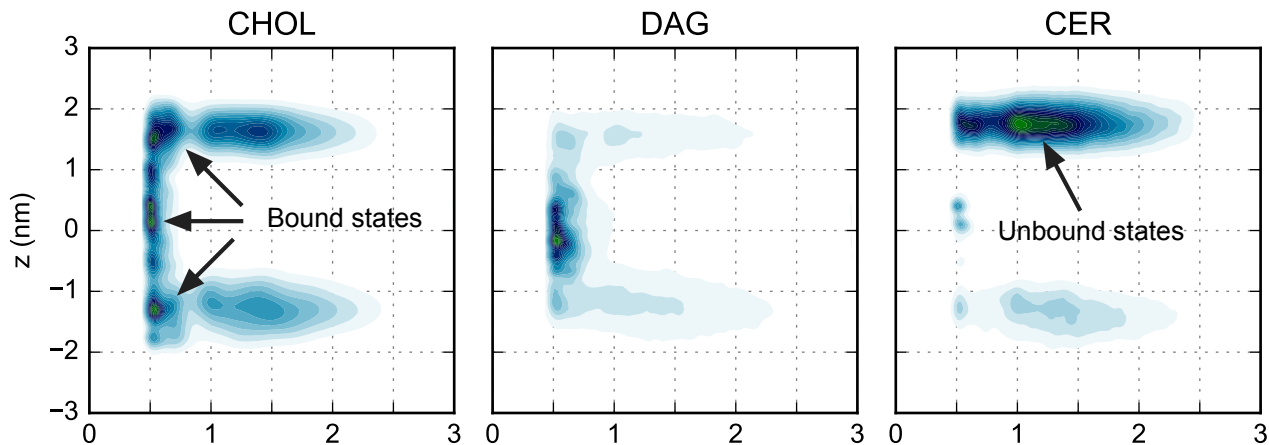

## ACTIVE MOR

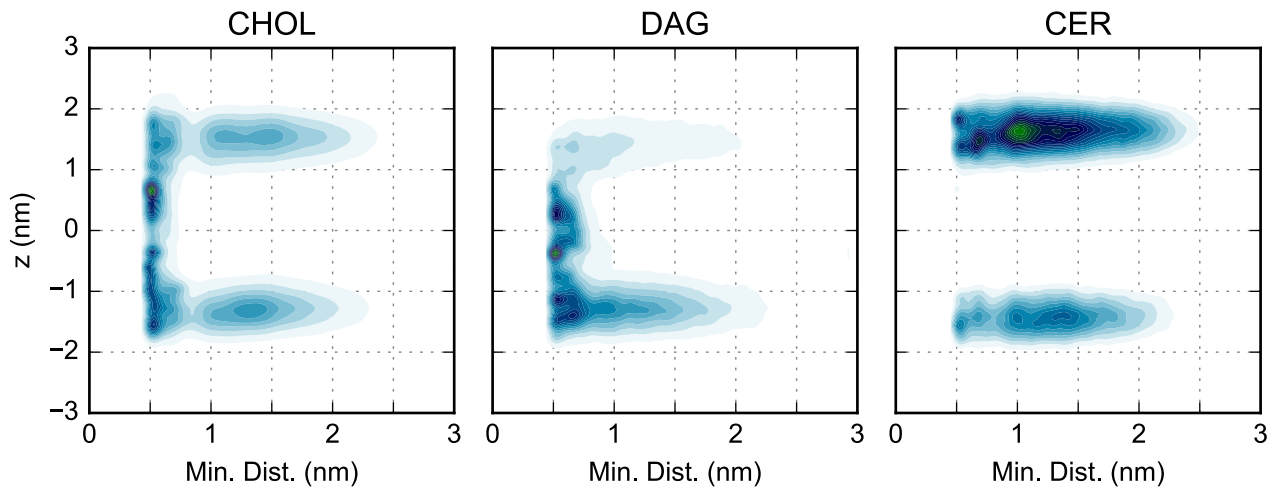

Supplement: S15 Fig — (PDF) [file pcbi.1005240.s018.pdf]

## DAG LIPIDS

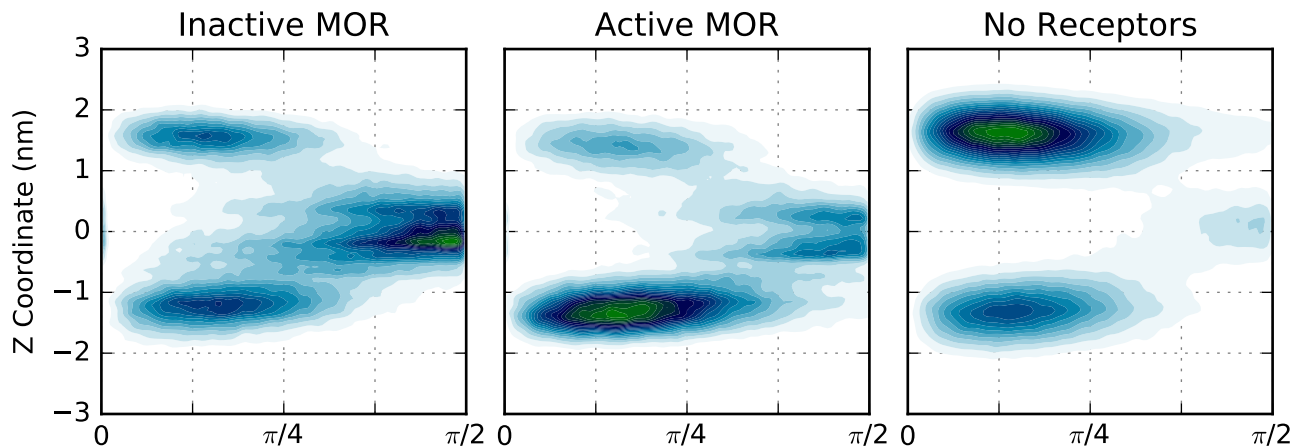

## CER LIPIDS

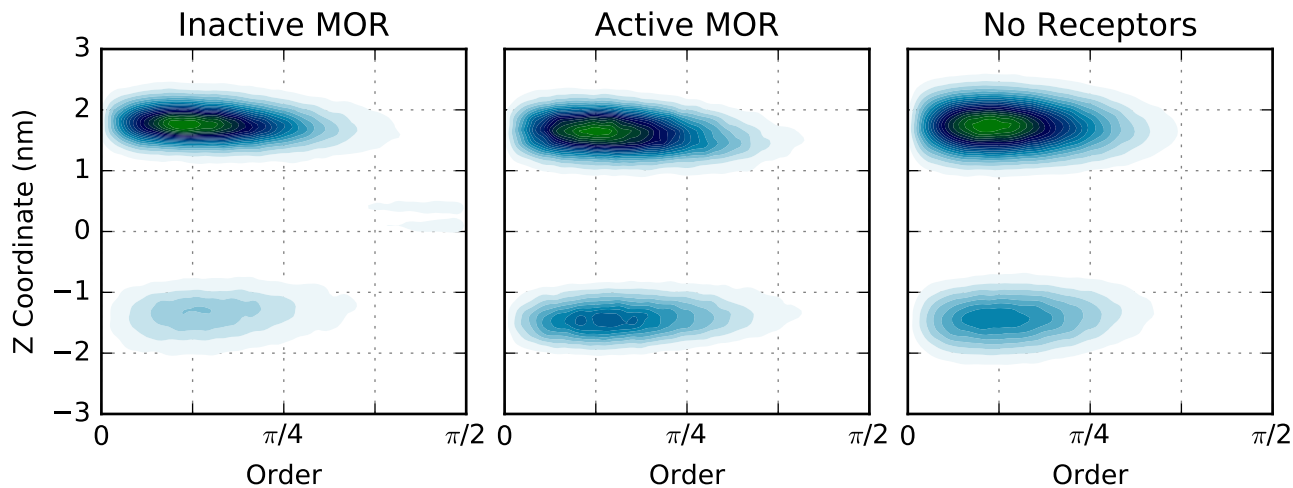

Supplement: S16 Fig — The data in the plots for the membranes with receptors are from the final 2 μs membrane equilibration in the simulations with high receptor density and the BB beads of the receptors fixed. (PDF) [file pcbi.1005240.s019.pdf]

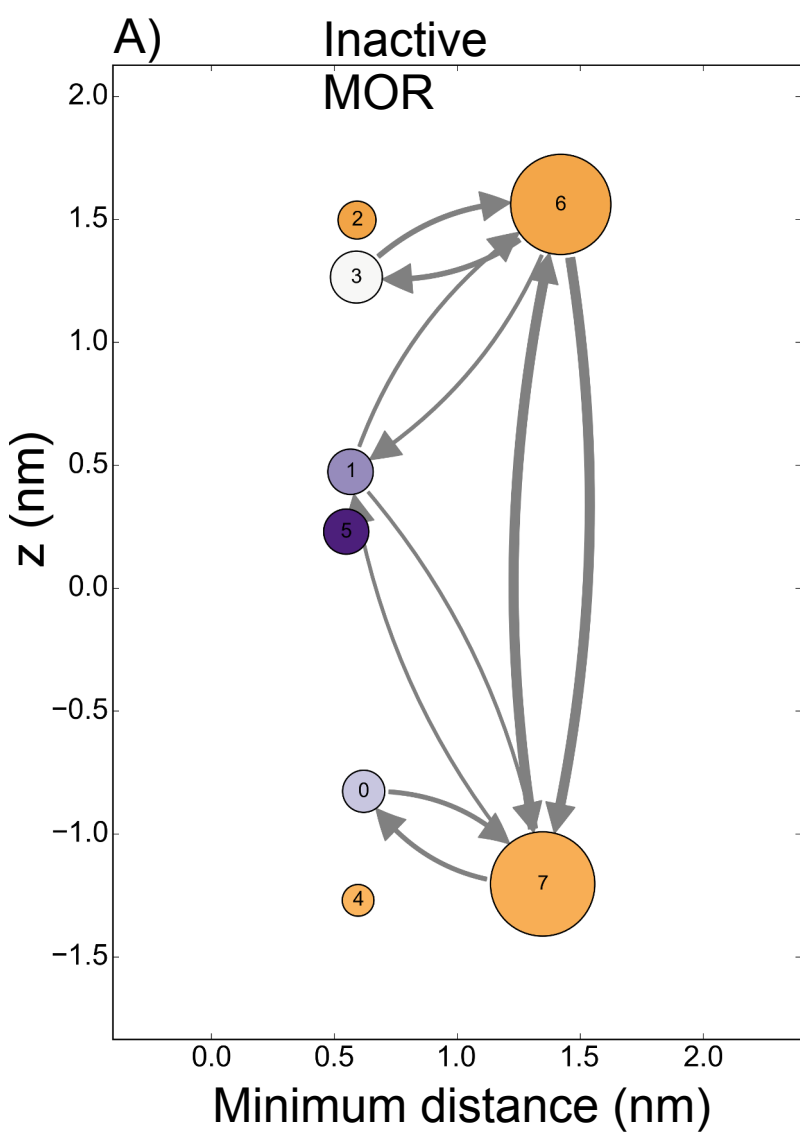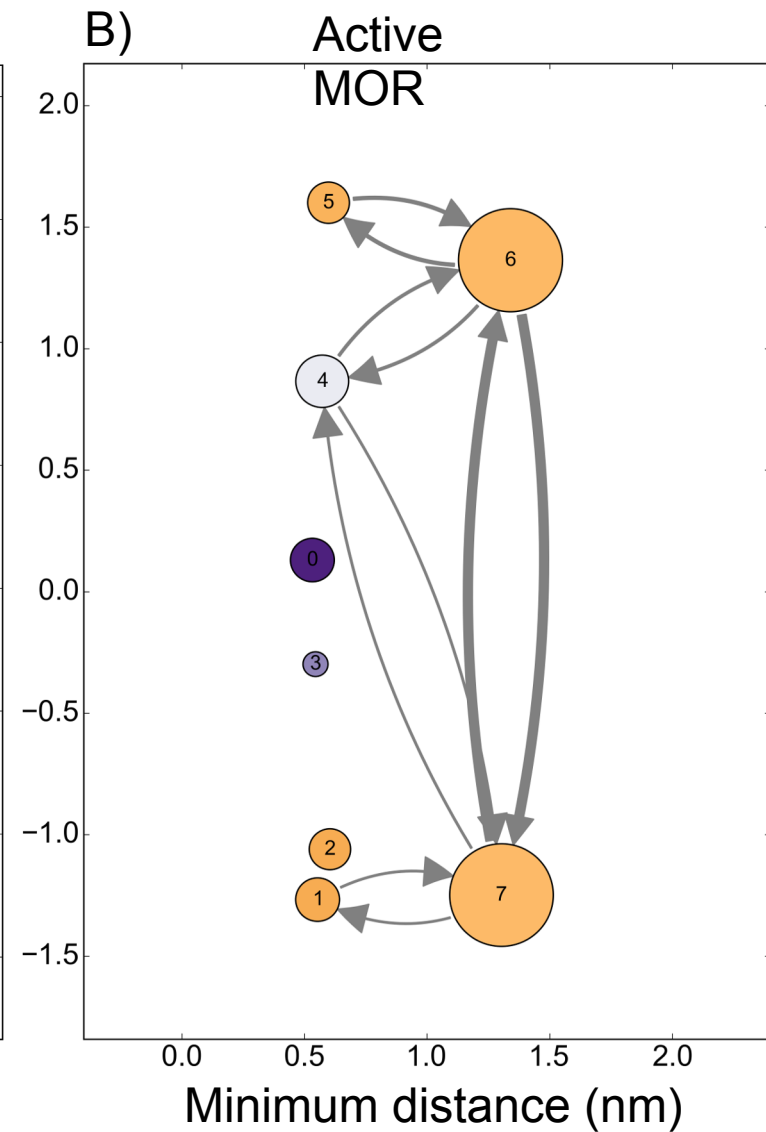

Supplement: S17 Fig — Kinetic network of the cholesterol movement in the z-direction as a function of the distance from the protein for the A) inactive and B) active MORs generated with the plot_network routine of pyemma using the final 2 μs of membrane equilibration of the simulations with high receptor density and the BB beads of the receptors fixed. The initial geometric clustering was performed using the contacts formed between a single cholesterol molecule and the residues of the protein. The five pathways with the highest fluxes are shown with the thickness of the arrows indicative of the relative flux. The size of the circle is proportional to the size of the state. The states are colored according to their average order with orange corresponding to a cholesterol oriented parallel to the membrane normal and dark purple representing a perpendicular orientation. (PDF) [file pcbi.1005240.s020.pdf]

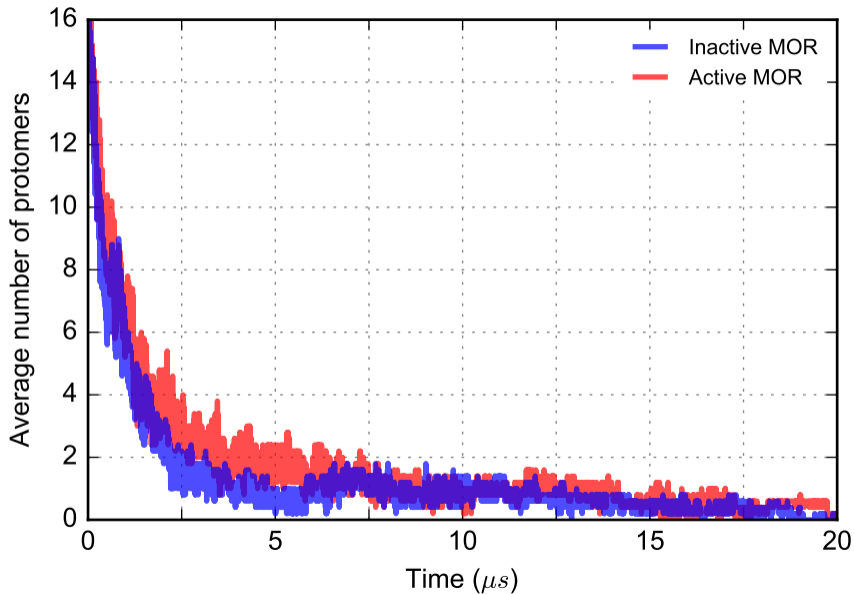

Supplement: S18 Fig — Values for the simulations with inactive or active receptors are reported in blue and red, respectively. (PDF) [file pcbi.1005240.s021.pdf]

A) TM1,2,H8/TM4

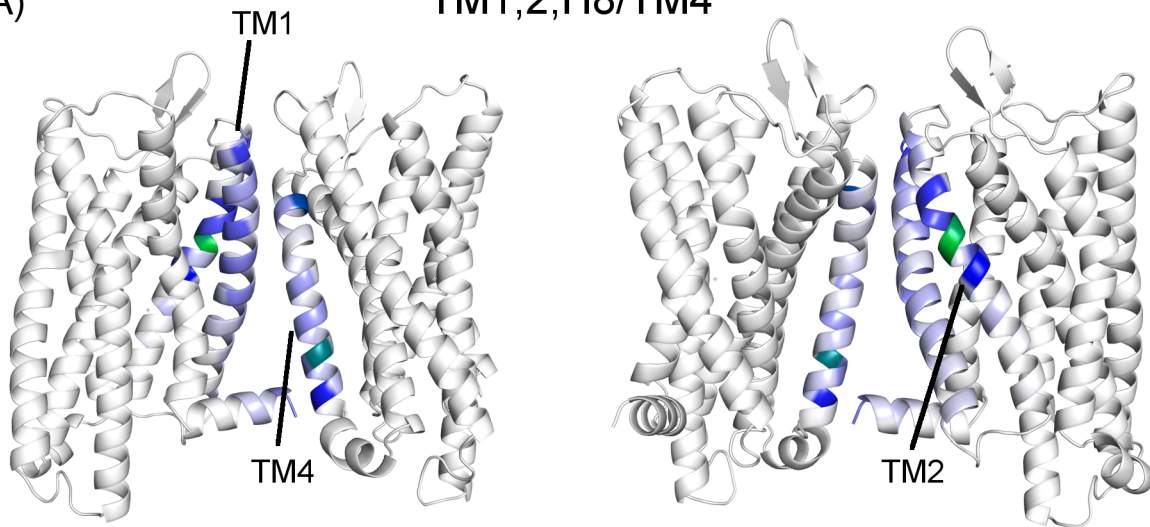

B) TM1,2,H8/TM1,2,H8

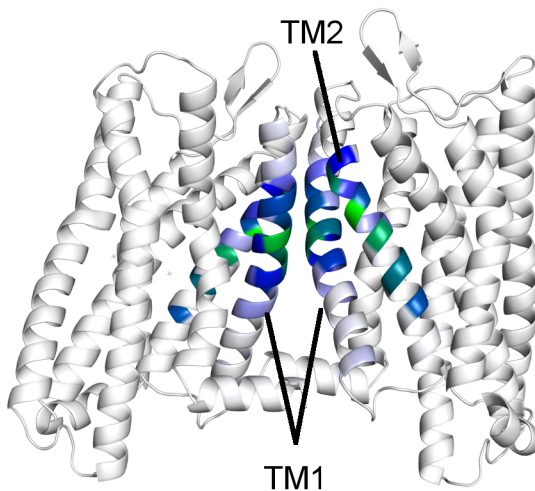

Supplement: S19 Fig — Model structures of the A) inactive/inactive MORs interacting at the TM1,2,H8/TM4 interface and B) active/active MORs interacting at the TM1,2,H8/TM1,2,H8 interface during the final μs of the high density simulations in which the BB beads of the receptors were kept fixed. Helices involved in the interface are colored by frequency of interaction with cholesterol (white to blue to green indicates low to high probability). (PDF) [file pcbi.1005240.s022.pdf]

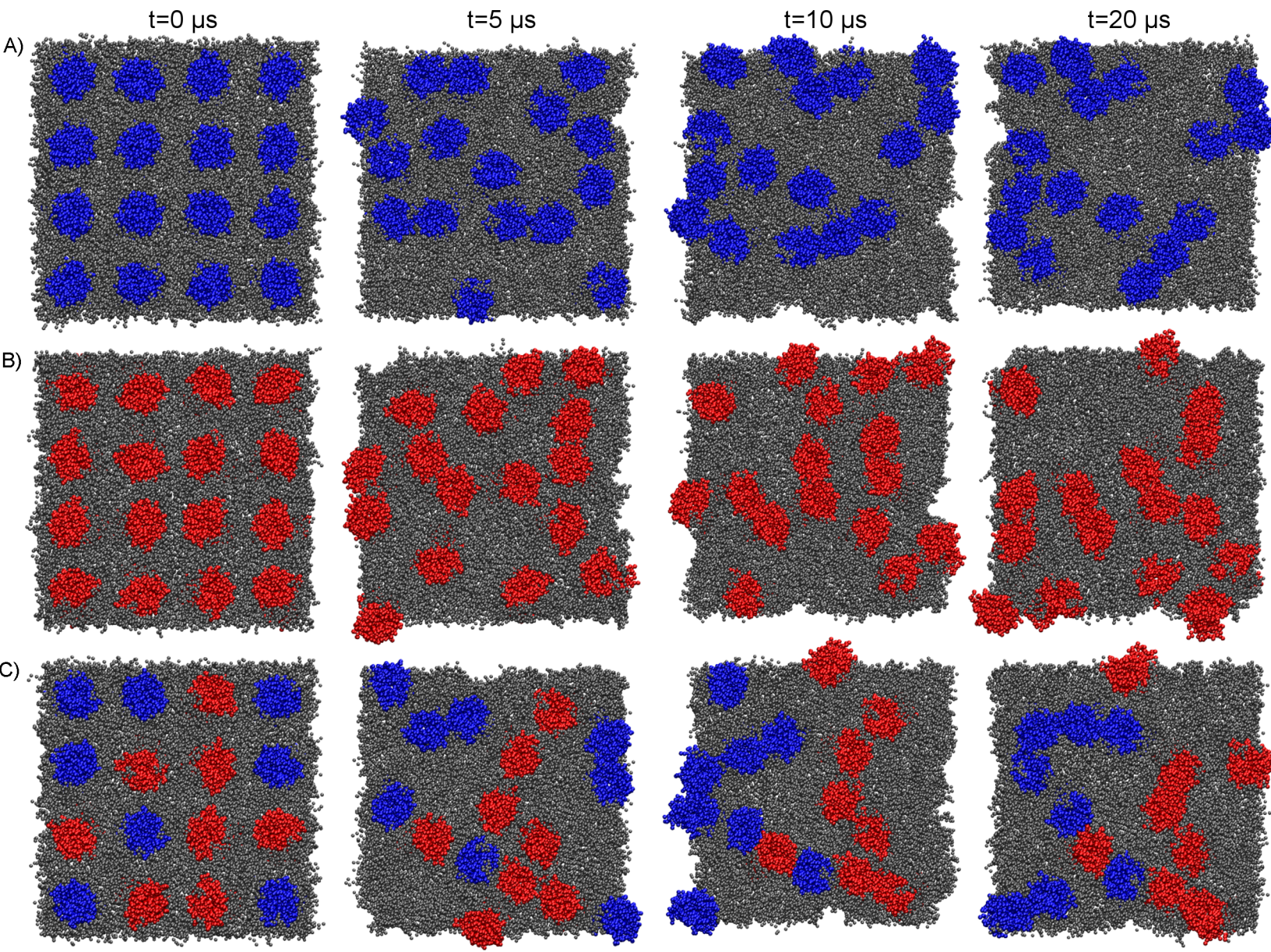

Supplement: S20 Fig — Snapshot of the production runs of the simulations with high receptor density at 0, 5, 10, and 20 μs for the A) inactive, B) active, and C) mixed arrays of MOR. The lipids are in grey, the inactive proteins in blue, and the active proteins are red. (PDF) [file pcbi.1005240.s023.pdf]
