# Supplementary material for: Impact of Lipid Composition and Receptor Conformation on the Spatio-temporal Organization of μ-Opioid Receptors in a Multi-component Plasma Membrane Model
Source: PLoS Comput Biol. 2016 Dec 13;12(12):e1005240. doi: 10.1371/journal.pcbi.1005240 (PMC5154498; doi:10.1371/journal.pcbi.1005240)
Supplement: S4 Fig — The data is averaged over all lipids, excluding the ones that flip-flop across the membrane (i.e. CHOL, CER, and DAG). For the order, a value of 0 (orange color) indicates a fully ordered lipid tail, and the larger the value, the more disordered the tail. The units of thickness are nm. In both cases, the white color is set to the average value of the simulations with the inactive receptors. The center of mass of the seven TM helices are indicated by the colored dots as follows: TMs 1 through 7 are colored in blue, red, grey, orange, yellow, green, and pink, respectively. (PDF) [file pcbi.1005240.s007.pdf]

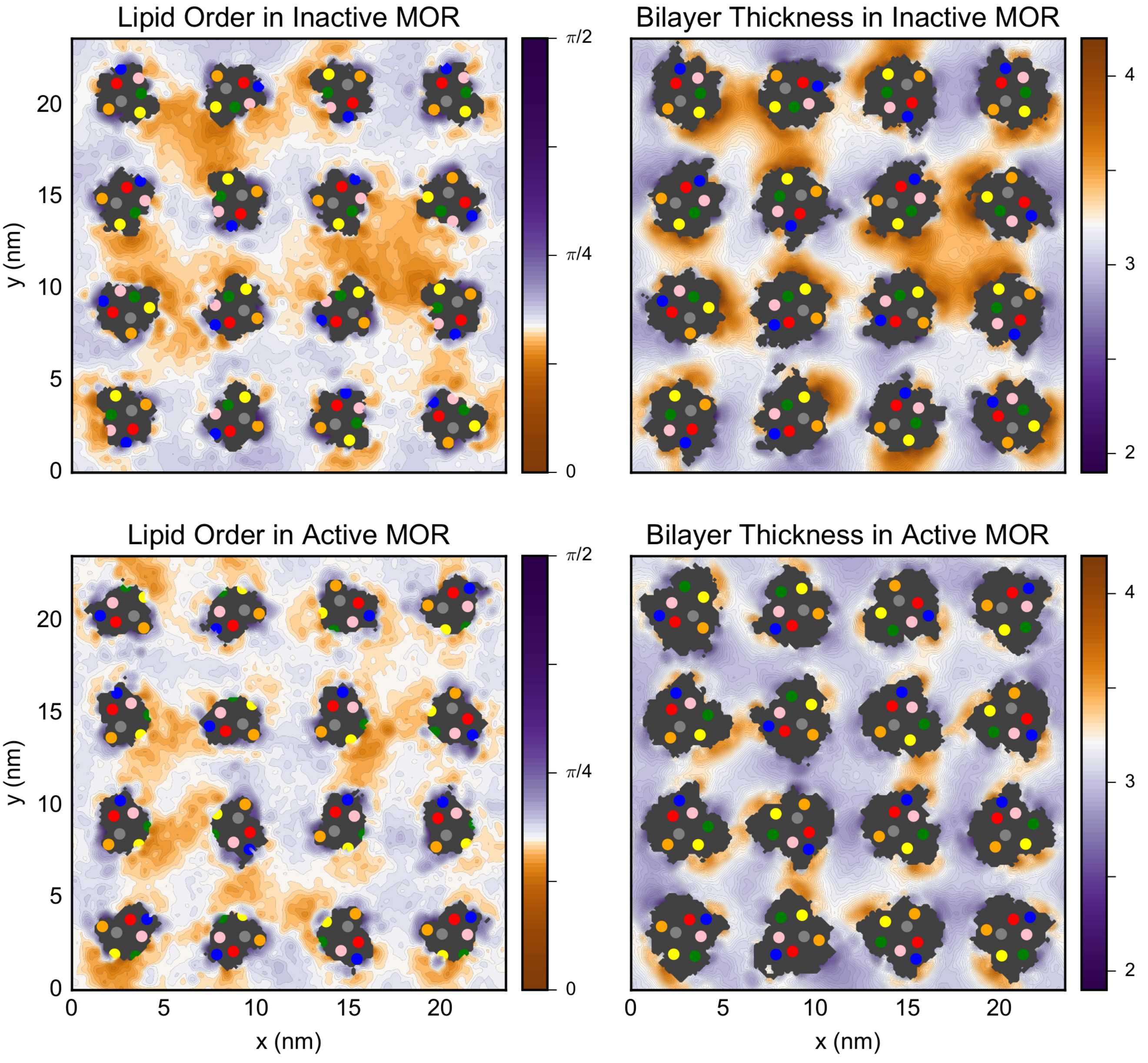

Lipid Order in Inactive MOR
Bilayer Thickness in Inactive MOR
Lipid Order in Active MOR
Bilayer Thickness in Active MOR
y (nm)
x (nm)
0
5
10
15
20
π/2
π/4
2
3
4
