# Supplementary material for: Impact of Lipid Composition and Receptor Conformation on the Spatio-temporal Organization of μ-Opioid Receptors in a Multi-component Plasma Membrane Model
Source: PLoS Comput Biol. 2016 Dec 13;12(12):e1005240. doi: 10.1371/journal.pcbi.1005240 (PMC5154498; doi:10.1371/journal.pcbi.1005240)
Supplement: S7 Fig — For the thickness, the average and standard error are indicated by a black line and a grey band, respectively. The overall average value of the order is indicated by red dashed lines in the bottom panels. The location of the center of mass of the helices is indicated by the vertical lines and TMs 1 through 7 are colored in blue, red, grey, orange, yellow, green, and pink, respectively. Because of the large tilt of TM3, its center of mass appears to the right of TM4. (PDF) [file pcbi.1005240.s010.pdf]

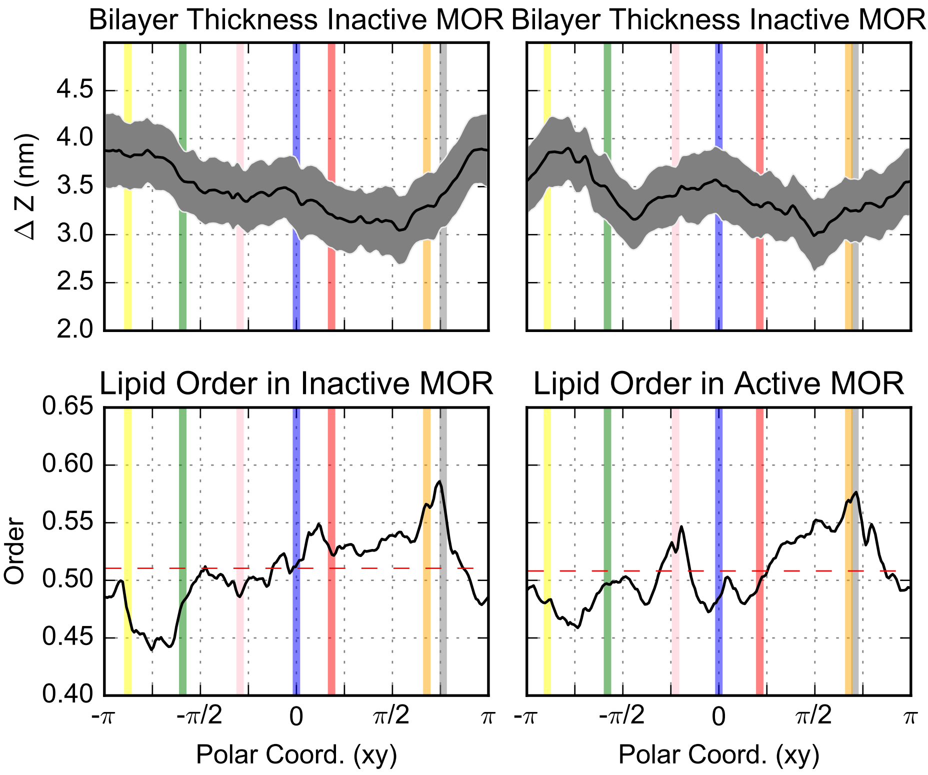

Bilayer Thickness Inactive MOR
Bilayer Thickness Inactive MOR
Δ Z (nm)
4.5
4.0
3.5
3.0
2.5
2.0
Lipid Order in Inactive MOR
Lipid Order in Active MOR
Order
0.65
0.60
0.55
0.50
0.45
0.40
-π
-π/2
0
π/2
π
Polar Coord. (xy)
Polar Coord. (xy)
