# Supplementary material for: Impact of Lipid Composition and Receptor Conformation on the Spatio-temporal Organization of μ-Opioid Receptors in a Multi-component Plasma Membrane Model
Source: PLoS Comput Biol. 2016 Dec 13;12(12):e1005240. doi: 10.1371/journal.pcbi.1005240 (PMC5154498; doi:10.1371/journal.pcbi.1005240)
Supplement: S8 Fig — For the thickness, the average and standard error are indicated by a black line and a grey band, respectively. The overall average value of the order is indicated by a red dashed lines in the bottom panels. The location of the center of mass of the helices is indicated by the vertical lines and TMs 1 through 7 are colored in blue, red, grey, orange, yellow, green, and pink, respectively. Because of the large tilt of TM3, its center of mass appears to the right of TM4. (PDF) [file pcbi.1005240.s011.pdf]

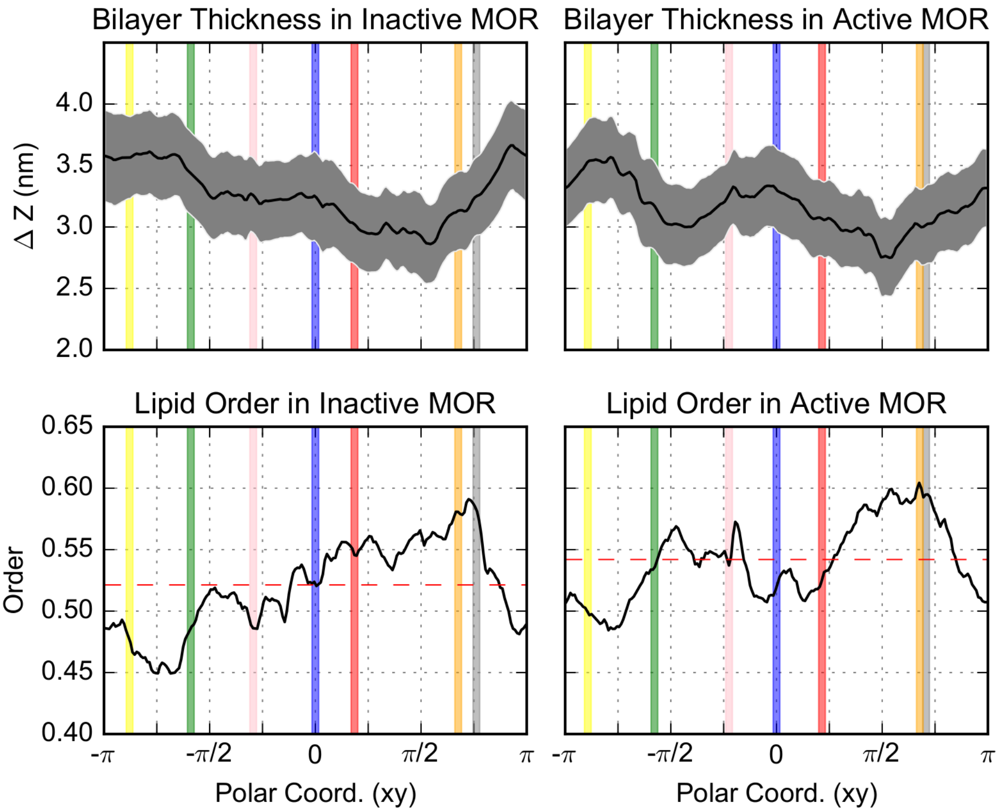
Bilayer Thickness in Inactive MOR
Bilayer Thickness in Active MOR
Δ Z (nm)
4.0
3.5
3.0
2.5
2.0
Lipid Order in Inactive MOR
Lipid Order in Active MOR
Order
0.65
0.60
0.55
0.50
0.45
0.40
-π
-π/2
0
π/2
π
Polar Coord. (xy)
Polar Coord. (xy)
